# Assessment for Direct Generation of Ocean Wave Energy: Dielectric Elastomer Generator and Dielectric Fluid Generator

**DOI:** 10.34133/research.1127

**Published:** 2026-02-10

**Authors:** Yao Zhang, Yutong Song, Teng Gao, Tianyi Zeng, Xin Dong, Xinyu Wang, Maozhou Meng, Richard Bucknall, Deborah Greaves

**Affiliations:** ^1^ Department of Mechanical Engineering, University College London, London WC1E 7JE, UK.; ^2^ School of Engineering, University of Southampton, Southampton SO17 8PX, UK.; ^3^ The Rolls-Royce UTC in Manufacturing and On-wing Technology, University of Nottingham, Nottingham NG8 1BB, UK.; ^4^ Offshore Renewable Energy Catapult, Blyth NE24 1LZ, UK.; ^5^ The School of Engineering, Computing and Mathematics, University of Plymouth, Devon PL4 8AA, UK.

## Abstract

Direct generation (DG) technologies—comprising dielectric elastomer generators (DEGs) and dielectric fluid generators (DFGs)—offer a promising paradigm for ocean wave energy conversion by integrating transduction mechanisms directly into wave-responsive materials. This assessment provides a comprehensive analysis of DG systems, outlining their working principles, recent material innovations, and comparative performance in harsh marine environments. We examine advancements in dielectric materials, including silicone-based and emerging nonsilicone elastomers, and discuss their influence on energy density, electromechanical efficiency, and environmental resilience. Comparative assessments highlight the advantages of DFGs in long-term durability and energy conversion under complex wave dynamics, while DEGs remain competitive due to their mechanical flexibility and scalable fabrication. The review concludes with a discussion of hybrid system integration, challenges in large-scale deployment, and a roadmap toward commercialization. By synthesizing current research trajectories, this article aims to accelerate the transition from laboratory-scale prototypes to deployable, cost-effective ocean energy harvesting solutions.

## Introduction

Wave energy, as a predictable and abundant renewable resource, holds immense potential to accelerate global decarbonization efforts. Theoretical estimates suggest that U.S. coastal waters alone could generate up to 2.64 trillion kWh annually [1], equivalent to 64% of the nation’s total utility-scale electricity generation in 2021 [2]. Despite this enormous potential, the commercial viability of wave energy remains constrained by technical challenges analogous to those faced by wind energy 3 decades ago. Similar to early wind turbine development, current wave energy converters (WECs) suffer from high levelized cost of energy (LCOE) stemming from structural complexity, maintenance burdens, and suboptimal energy conversion efficiency in harsh marine environments [3].

In response to these challenges, direct generation (DG) technologies represent a transformative approach by fundamentally rethinking the architecture of WECs. Unlike traditional designs that rely on complex, rigid, and maintenance-intensive power take-off (PTO) systems—such as hydraulic rams or mechanical gearboxes—DG technology integrates the energy conversion mechanism directly into the device’s flexible structure. This design philosophy, championed by industry leaders like Wave Energy Scotland [4], offers a direct solution to the core bottlenecks of cost and reliability. By eliminating bulky intermediate components, it enables a new class of lightweight, flexible architectures that reduce non-energy-generating mass by over 50% [5]. This integration also enhances survivability by replacing a single point-of-failure PTO with a distributed, modular generation system, improving fault tolerance. Furthermore, the reliance on polymers opens the potential for applying scalable, cost-effective mass-manufacturing processes, a marked departure from the bespoke fabrication of conventional WECs. This approach, combined with inherent low-frequency adaptability (0.1 to 0.3 Hz), directly addresses the critical challenges of structural complexity and high maintenance burdens that have historically plagued the wave energy sector [6].

DEGs leverage deformable polymer matrices, such as silicone, where mechanical stretching induces capacitance variations to generate electrical energy. The biomimetic nature of dielectric elastomers (DEs) enables muscle-like adaptability in wave energy conversion [7]. Silicone-based elastomers are favored for their high permittivity ($\epsilon_{r}∼3.0$) and resilience to cyclic loading, enabling efficient energy capture across diverse wave conditions [8]. Notably, natural rubber (NR) demonstrates superior energy capture efficiency at strains below 15% due to its unique hyperelastic behavior [8]. Recent advancements in bistable electroactive polymers further enhance load-bearing capacity under extreme wave loads (>5 m wave height) through snap-through instability mechanisms [7].

In contrast, DFGs utilize pressurized dielectric fluids subjected to wave-induced pressure fluctuations, allowing for adaptive tuning to variable wave frequencies. Crucially, this design philosophy eliminates the complex mechanical linkages that are often the primary source of failure and high maintenance costs in conventional PTOs. This enables highly integrated and potentially more robust architectures, such as air-filled bladders or tethered carpets, achieving power-to-weight ratios comparable to biological muscle (~0.1 kW/kg) [7]. Both DG approaches thus exemplify the potential of material-driven innovation to overcome traditional WEC limitations.

Recent research has prioritized synergistic advancements in materials, system integration, and manufacturing to enhance DG performance. Wave energy represents a vast untapped renewable resource. Global theoretical wave power potential is estimated at approximately 2.11 ± 0.05 TW [9], with regional assessments revealing substantial exploitable resources: South America’s west coast offers 324 GW, Australia and New Zealand combined provide 574 GW, western North America contributes 207 GW, and northern/western Europe accounts for 286 GW [9]. Despite this multi-terawatt potential, wave energy conversion technologies including dielectric generators (DGs) remain in early deployment stages. When restricting attention to regions with mean wave power densities above about 5 kW/m, the global theoretical wave energy resource is on the order of 29,500 TWh per year [10,11]. In contrast, techno-economic studies typically estimate the practically or economically exploitable resource in the range of 2,000 to 4,000 TWh per year [11]. For context, global electricity demand in 2022 was approximately 28,500 TWh, implying that the practical wave resource alone is of the same order as roughly 7% to 14% of current annual electricity consumption [12].

Material innovations have overcome traditional dielectric limitations through barium titanate-doped silicone composites, achieving relative permittivity ($\epsilon_{r}$) values exceeding 12 while reducing viscoelastic losses by 30% compared to conventional polydimethylsiloxane (PDMS) [7]. Concurrently, self-healing ionic networks in silicones enable autonomous repair of microcracks, maintaining over 95% capacitance retention through 10,000 damage–repair cycles in a laboratory setting [13]. System architectures now incorporate self-powered modules with integrated charge pumps, allowing fully autonomous operation in remote offshore deployments without grid dependencies [5]. This is complemented by fault-tolerant designs using segmented DEG membranes, where redundant electrical interconnects achieve 99.2% system reliability through localized failure isolation [6]. Manufacturing scalability has been revolutionized through roll-to-roll production of sub-100-μm dielectric films, cutting unit costs by 68% while maintaining sub-millimeter tolerances [8]. Further durability enhancements include ultraviolet (UV)-resistant coatings that extend service life by 40% in tropical marine environments [3]. These coordinated breakthroughs position DG technologies as viable solutions for both distributed sensor networks and utility-scale wave farms.

Despite progress, critical challenges hinder the full-scale deployment of DG technologies. Material fatigue remains a primary concern, with elastomers currently enduring approximately 10^6^ loading cycles—far below the 10^8^ cycles required for a 25-year lifespan [14]. This limitation is exacerbated by electrode degradation: Compliant ionic gel electrodes, while enabling >300% strain through ion migration mechanisms, exhibit rapid performance decay after merely 10^5^ cycles [7]. Environmental sustainability poses additional multifaceted risks. Long-term seawater exposure erodes silicone membranes, releasing microplastics at rates of 2.8 mg/m^2^/day—equivalent to 1.02 kg annually per 1-MW WEC installation [3]. Historically, the marine energy sector has also struggled with ecosystem contamination from lubricants and anti-biofouling agents, adding environmental concerns to the existing technical and economic barriers [1]. Compounding these issues, recycling thermoset elastomers demands energy-intensive pyrolysis at 800 °C, emitting 3.2 kg CO_2_e per kilogram processed [5]. Furthermore, maintaining electrical efficiency at ultralow wave frequencies (e.g., 0.14 Hz) requires innovative power electronics to prevent dielectric breakdown under high-voltage conditions [4]. These interdependent barriers underscore the need for interdisciplinary research and development to bridge laboratory innovations and field-ready solutions.

This article aims at assessing the state-of-the-art in DG technologies for wave energy conversion. DE Materials Investigation and Comparison categorizes DEs into silicone-based and nonsilicone-based materials, analyzing their electromechanical properties. Dielectric Elastomer Generators and Dielectric Fluid Generators delve into the working principles and performance metrics of DEGs and DFGs, respectively. A comparative analysis in Comparative Analysis of DEGs and DFGs evaluates their suitability for offshore applications based on fatigue resistance, efficiency, and scalability. Finally, Recent Advances and Future Directions explores emerging materials and hybrid architectures, concluding with a roadmap for commercialization. By synthesizing theoretical insights and practical advancements, this review aims to accelerate the transition from prototype validation to industrial adoption.

## DE Materials Investigation and Comparison

The force–displacement plot of DEG shows the input mechanical energy ($W_{\text{mech}}$) in one cycle (area I–II–III–IV), while the voltage–charge plot reveals 4 failure modes: electromechanical instability (EMI), electrical breakdown (EB), mechanical rupture (MR), and loss of tension (LT). DEG configurations include equibiaxial, pure shear, diamond, cone, and circular diaphragm types. The operating cycle consists of 2 isopotential phases (deflection and inflation) and 2 isochoric phases (priming and discharging), where *V* represents electrode voltage and Ω represents the fluid volume.

### DE materials: Fundamentals and classification

**Fig. 1.**
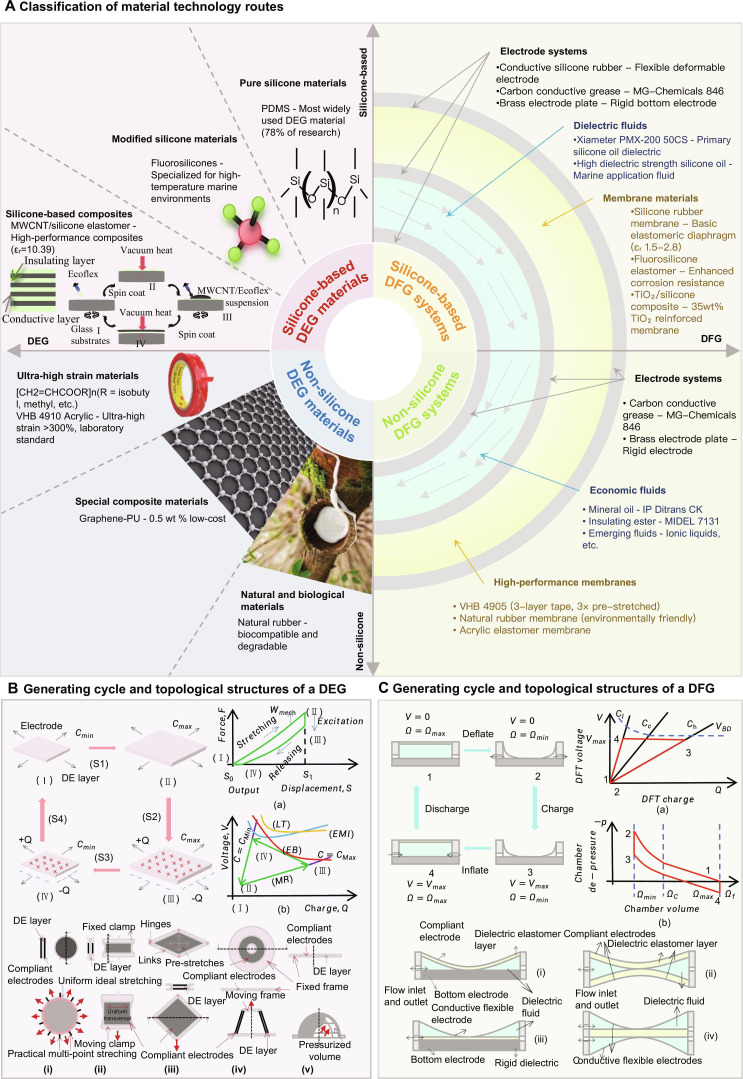
Classification of material technology routes for DG systems. (A) Material technology classification. (B) DEG generating cycle and topological structures. (C) DFG generating cycle and topological structures. (B) and (C) are based on the concepts described in [5,43,103].

DG technologies leverage advanced material science to achieve efficient electromechanical energy conversion. Figure 1 provides a comprehensive classification of material technology routes, working principles, and system architectures for both dielectric elastomer generators (DEGs) and dielectric fluid generators (DFGs). This classification framework serves as a foundation for understanding the material selection criteria and performance trade-offs in wave energy harvesting applications. Figure 1 presents a comprehensive overview of the material technology landscape for DG systems. The classification shows the hierarchical structure of dielectric materials from pure silicone to specialized composites, electrode systems, and dielectric fluids. Figure 1A illustrates the relationship between silicone-based materials (78% of research focus), nonsilicone alternatives, and emerging composite technologies. Figure 1B demonstrates the 4-step electromechanical conversion process with charge–voltage relationships for DEGs. Figure 1C shows the thermodynamic cycle with pressure–volume relationships and fluid-based energy conversion mechanisms for DFGs.

DEs are electroactive polymers that undergo mechanical deformation under applied electric fields. Their actuation mechanism is governed by Maxwell stress (σ) [8]: $\sigma =\varepsilon_{0}\varepsilon_{r}E^{2}$, where $\varepsilon_{0}$ is vacuum permittivity, $\varepsilon_{r}$ is the relative dielectric constant, and *E* is the electric field.

DE materials are broadly categorized into 3 classes based on molecular structure and application scope (Fig. 1) [15]. Silicone-based elastomers are dominant in research (78% of publications) due to balanced $\varepsilon_{r}$ (2.8 to 6.2), low *Y* (0.1 to 2 MPa), and thermal resilience (−100 to −300 °C) [16]. Representative types include PDMS and fluorosilicones (detailed in DE Materials Investigation and Comparison). Nonsilicone polymers include several subtypes: Acrylics (e.g., VHB 4910) offer ultra-high strain (>300%) but suffer from viscoelastic hysteresis; polyurethanes (PUs) feature tunable modulus (1 to 100 MPa) for precision actuation [17]; NR is biodegradable and low-cost [5,18]; and fluoropolymers [e.g., polyvinylidene fluoride (PVDF)] provide high $\varepsilon_{r}$ (10 to 12) with piezoelectricity [15], yet become brittle below −40 °C (analyzed in Dielectric Elastomer Generators). Composites represent hybrid systems [e.g., silicone/multi-walled carbon nanotube (MWCNT) and PU/BaTiO_3_] engineered to overcome intrinsic trade-offs.

The optimal DE material depends on application priorities. Table 1 summarizes the material selection criteria for different application requirements.

**Table 1.** Material selection criteria for dielectric elastomers

Application criteria	First choice	Second choice	Third choice
High strain/energy density	Acrylics	Silicones	PVDF
Fatigue resistance	Silicones	Natural rubber	Acrylics
Extreme environments	Fluorosilicones	PVDF	Natural rubber
Biocompatibility	Natural rubber	Silicones	Acrylics

This classification provides a roadmap for selecting DE materials, with silicone-based elastomers emerging as the default choice for most scenarios. Their molecular design flexibility and performance stability are systematically analyzed in the following subsections. Silicone-based elastomers have emerged as the cornerstone of DE research due to their unique combination of elasticity, thermal stability, and dielectric properties. These materials exhibit superior fatigue resistance, maintaining consistent performance over thousands of stretching cycles—a critical requirement for applications like energy harvesting and soft robotics. Their high Poisson’s ratio (~0.5) enables incompressible behavior, allowing strain amplification without volume change, which is essential for maximizing electromechanical coupling efficiency. Additionally, silicone elastomers operate reliably across a broad temperature range (−100 to 200 °C), making them adaptable to extreme environments such as aerospace or deep-sea applications.

A key advantage of silicone-based materials lies in their chemical versatility. Functional modifications, such as fluorine substitution or the introduction of dipole structures (e.g., COOH or COOCH_3_ groups), can significantly enhance dielectric properties without compromising elasticity. For example, fluorine-substituted silicone elastomers demonstrate improved insulation resistivity and self-repairing capabilities through heat treatment, addressing common failure modes like electrical treeing. However, these benefits come with trade-offs: Complex synthesis processes and potential mechanical degradation at high fluorine content limit scalability.

In contrast, nonsilicone materials like PVDF and NR face inherent challenges. PVDF, while offering high piezoelectricity and chemical stability, suffers from reduced ductility at low temperatures and viscoelastic losses under cyclic loading. NR, though cost-effective and biocompatible, struggles with limited corrosion resistance in harsh environments. These limitations underscore why silicone-based materials remain the default choice for most DE applications.

### Silicone-based materials: Innovations and limitations

Recent advancements in silicone-based materials have focused on systematically optimizing dielectric and mechanical properties through innovative composite engineering approaches. Beginning with fluorine-substituted silicone elastomers, researchers have successfully enhanced DC resistivity, breakdown strength, and dielectric constants [19] while simultaneously improving plasticity and tear resistance through stronger chemical bonding. This foundation has paved the way for more sophisticated architectures, including liquid–solid interpenetrating structures where hydroxyl silicone oil is incorporated into silicone rubber foam. When reinforced with TiO_2_ nanoparticles (typically at 35 wt %), these composites achieve remarkable increases in dielectric constant and electromagnetic performance [16], although excessive filler loading introduces dielectric losses that manifest as heat rather than useful elastic energy.

The manipulation of molecular structure through dipole engineering represents another promising direction, where carboxyl, methoxycarbonyl, and hydroxyl modifications to polymethylvinylsiloxane (PMVS) yield varying improvements in tensile strength and dielectric properties [20]. Notably, hydroxyl-modified PMVS demonstrates superior tensile strain under high electric fields (15 kV/mm), establishing critical structure–property relationships for DEGs. Perhaps the most significant breakthrough has been the development of 3-dimensionally (3D) separated MWCNT networks within silicone matrices [21]. These architectures strategically limit charge transport between adjacent nanotubes while achieving dielectric constants of approximately 10.39 at minimal filler content (0.6 wt %), enabling actuation strains of 11.61% at field strengths of only 10.1 V/μm.

Further refinements have led to laminated MWCNT/silicone composites that push performance boundaries to 20.3% actuation strain with excellent cyclic stability over 100 actuation cycles [22]. Despite their impressive electromechanical sensitivity, these materials face practical implementation challenges including high operational voltages exceeding 100 kV/mm and susceptibility to leakage currents as conductive particle loading approaches the percolation threshold. Functionalized polyoctahedral siloxanes (POSS) and nanospring carbon nanotubes (NS-CNTs) represent the current frontier [23], with the latter achieving a balance of dielectric constant (4.6), minimal viscoelastic loss (0.03 mechanical loss coefficient), and exceptional ductility (270% strain at fracture). DEGs constructed from these advanced materials demonstrate progressively increasing voltage output with strain (from 8.8 V at 33% to 14.5 V at 66%), confirming the direct relationship between engineered dielectric properties and energy conversion efficiency. While challenges remain in optimizing compatibility, preventing aggregate formation, and determining ideal filler concentrations, these silicone-based composites collectively represent a significant advancement toward practical, high-performance DEG applications.

### Nonsilicone-based materials: Niche applications and trade-offs

Nonsilicone dielectric materials offer compelling alternatives for specialized applications where conventional silicones fall short (Tables 2 and 3). UNDE’s stable 10-Hz performance significantly outperforms silicone’s 5-Hz limit, making it ideal for high-frequency actuation requirements. In simulated marine environments, CNT–Al_2_O_3_ composites demonstrate remarkable durability with 78% lower corrosion rates compared to PDMS [24] while also providing 65% increased dielectric constant and 40% enhanced breakdown strength. For energy-intensive applications, ZrO_2_–PDMS achieves triple the energy storage capacity of standard silicones [25] with 1.2J/cm^3^ density and 150% area strain, despite challenges with 0.8 wt % aggregation threshold and 300% increased modulus. Cost-sensitive implementations benefit significantly from graphene–PU formulations [17], which reduce electrode costs by 62% through optimized filler distribution at minimal 0.5 wt % loading efficiency, though with 15% increased leakage current. For extreme deformation scenarios, VHB elastomers maintain 89% efficiency at 400% strain (compared to silicone’s 72%) under laboratory test conditions, achieving 500% total strain capacity at competitive $0.35/cm^2^ cost despite 30% hysteresis loss.

**Table 2.** Comparative analysis of silicone-based dielectric materials

Material type	Key advantages	Limitations	Performance
Fluorinated silicone	High insulation, self-healing, tear resistance	Undocumented	$\varepsilon_{r}$↑, breakdown↑
TiO_2_/silicone Foam	Enhanced $\varepsilon_{r}$ (35 wt %), EM performance	High TiO_2_ → loss↑	FOM index↑
Dipole-modified	Tunable Y/$\varepsilon_{r}$, high strain	Dipole >15% → performance ↓	*Y* = 0.21 MPa
DEG silicone	Fatigue/corrosion resistance	Extreme condition aging	15 kV/mm efficiency
3D-MWCNT	$\varepsilon_{r}$ = 10.39, low loss	High loading breakdown	11.6%@10.1 V/μm
Laminated MWCNT	20.3% strain, stable cycles	Requires >100 kV/mm	High sensitivity
NS-CNTs	$\varepsilon_{r}$: 3.2–8.6, strain: 100–250	Strength trade-off	14.5V@66%

**Table 3.** Comparative analysis of nonsilicone dielectric materials

Material	Key advantages	Limitations	Performance	Applications
UNDE (BNNS)	Stable 0.1–10 Hz operation, high folding endurance, high-frequency actuation	Complex fabrication, high activation threshold, limited scalability	Strain 8–10% [144] @8 kV, 10 Hz	Flexible actuators, high-frequency apps
CNT–Al_2_O_3_	$\varepsilon_{r}$ up 65%, breakdown strength +40%, corrosion rate −78%	Field distribution issues, low dispersion limit, complex preparation	23 kV/mm BD strength	Marine, high-voltage applications
ZrO_2_–PDMS	Energy: 1.2 J/cm^3^, 150% area strain, high energy storage	Low aggregation threshold, modulus +300%	12.8%/kV actuation	Energy harvesters, flexible electronics
Graphene–PU	0.5 wt % loading, 80% cost reduction, high mechanical strength	Increased leakage current, limited cycle stability, poor scalability	18.4 FOM index	Flexible electronics, sensors
VHB elastomer	500% strain capacity, low cost, high strain performance	30% hysteresis loss, low temperature limit, poor in extreme temperatures	Energy density: 560 (mJ·g^−1^) [34]	Wearable sensors, marine energy
PVDF	Low loss tangent, high dielectric strength, piezoelectric properties	Low-temperature embrittlement, complex crystallization, limited flexibility	12 pC/N response	High-voltage actuators, energy harvesting
PUA	High tensile strength, adjustable modulus, tunable structure	Property trade-offs, reduced lifetime, variable properties	Strain 590–600% [145]	Flexible electronics, biomedical
Natural rubber	Excellent tear resistance, high ductility, low production cost	Needs marine protection, lower electrical properties, environmental degradation	High reversible deformation	Motion sensing, biomedical
PEEK	Excellent fatigue life, biocompatibility, bone-like modulus	Thermal stress issues, high melting point, complex processing	Nonlinear strain response	Bone repair, DEG applications

Light-curing polyurethane acrylate (PUA) offers distinctive advantages with its excellent flexibility and controllable mechanical properties [26]. PUA elastomers can achieve a maximum tensile yield strength of 389% while maintaining a Young’s modulus below 1.2 MPa through precise formulation control. By adjusting polymer chain length and functional endpoints, researchers can engineer 2 different crosslinking networks—decentralized points or locally concentrated regions—optimizing performance for specific applications. PUA demonstrates balanced electro-mechanical properties, achieving 13.88% maximum tensile strain at 45.41 V/μm breakdown electric field strength. However, these materials sometimes present a contradictory relationship between dielectric constant and tensile strain, and their breaking strength and lifetime can deteriorate under high electric field conditions due to chain length limitations and crosslink density variations.

Poly(vinylidene fluoride) (PVDF) represents another significant alternative with its unique semicrystalline piezoelectric properties [15]. PVDF incorporates 4 distinct crystalline phases—α, β, γ, and δ—resulting in parallel dipoles and a net dipole moment that enhances its electrical performance. Its high dielectric constant, attributable to fluorine atom electronegativity, combines with a high DC breakdown field and remarkably low loss tangent (0.02) to create a material well-suited for rapid deformation applications. Mechanically, PVDF offers high density, Young’s modulus, and strength, coupled with minimal moisture absorption for improved stability in humid environments. As a viscoelastic polymer, PVDF induces substantial driving strains under electric fields, enabling efficient mechanical–electrical energy conversion. Its light weight, flexibility, and chemical stability make it particularly valuable for DEG systems, though its performance may decline at temperatures below −20 °C due to embrittlement.

NR continues to demonstrate significant potential for DEG applications [18], particularly in human motion sensing and biomedical sensors. Its exceptional elasticity and ductility enable large, reversible deformations without rupture, ensuring stable long-term operation. Superior tear resistance reduces damage susceptibility during electric field deformation, enhancing overall durability. NR’s excellent electrical insulation properties minimize external electromagnetic interference, improving energy performance in DEG systems. Despite relatively modest electrical properties compared to synthetic alternatives, NR remains highly competitive due to its cost-effectiveness, with ongoing research focusing on multi-objective optimization to address the trade-off between convertible energy and operational lifetime.

Polyetheretherketone (PEEK) emerges as a multifunctional material with applications spanning both bone repair and DEG systems [27]. Its 2-phase semicrystalline structure exhibits exceptional viscoplastic behavior with nonlinear strain rate sensitivity and temperature dependence [28]. In bone repair applications, PEEK’s biocompatibility and elastic modulus—remarkably similar to human bone–position it as an ideal candidate, particularly when fabricated using advanced 3D printing technologies. For DEG implementations, PEEK’s excellent fatigue life enables multiple deformation cycles without failure, while its outstanding elastic modulus and ductility provide the flexibility required for electric field-induced deformation, facilitating efficient mechanical-to-electrical energy conversion.

The future of nonsilicone dielectrics is being shaped by several innovative approaches. Hybrid architectures combining UNDE’s frequency stability with PVDF’s piezoelectric properties (12 pC/N response) create synergistic performance profiles suitable for self-sensing actuators. Material processing has advanced significantly with machine learning (ML)-optimized dispersion protocols achieving 93% homogeneity for ZrO_2_ nanoparticles, substantially improving reliability and performance consistency. Biomimetic strategies, particularly graphene alignment techniques inspired by nacre microstructure, offer promising pathways to overcome current limitations in mechanical resilience while preserving electrical properties. These emerging solutions represent the cutting edge of dielectric material development, addressing the performance gaps that have traditionally limited nonsilicone alternatives while capitalizing on their unique advantages for specialized applications requiring extreme strain capacity, marine durability, or high-frequency response.

Nonsilicone elastomers carve out niche roles where silicone falls short. PU, for example, excels in applications demanding high strain-at-break and electromagnetic interference resistance. Its adaptability to controlled deformation makes it ideal for flexible electronics and biomedical devices. Graphene–epoxy nanocomposites, meanwhile, leverage graphene’s exceptional fracture toughness and thermal conductivity for DEGs operating in interference-prone environments. Despite these strengths, high production costs and scalability issues hinder widespread adoption.

NR remains a compelling option for low-cost, eco-friendly systems. Its biocompatibility and tear resistance suit wearable sensors and marine energy harvesters. However, performance degradation in acidic or alkaline environments necessitates protective coatings, adding complexity. PVDF’s piezoelectricity offers unique advantages in high-energy-density applications, but its brittleness at low temperatures restricts use in subzero conditions.

### Comparative analysis and future directions

**Fig. 2.**
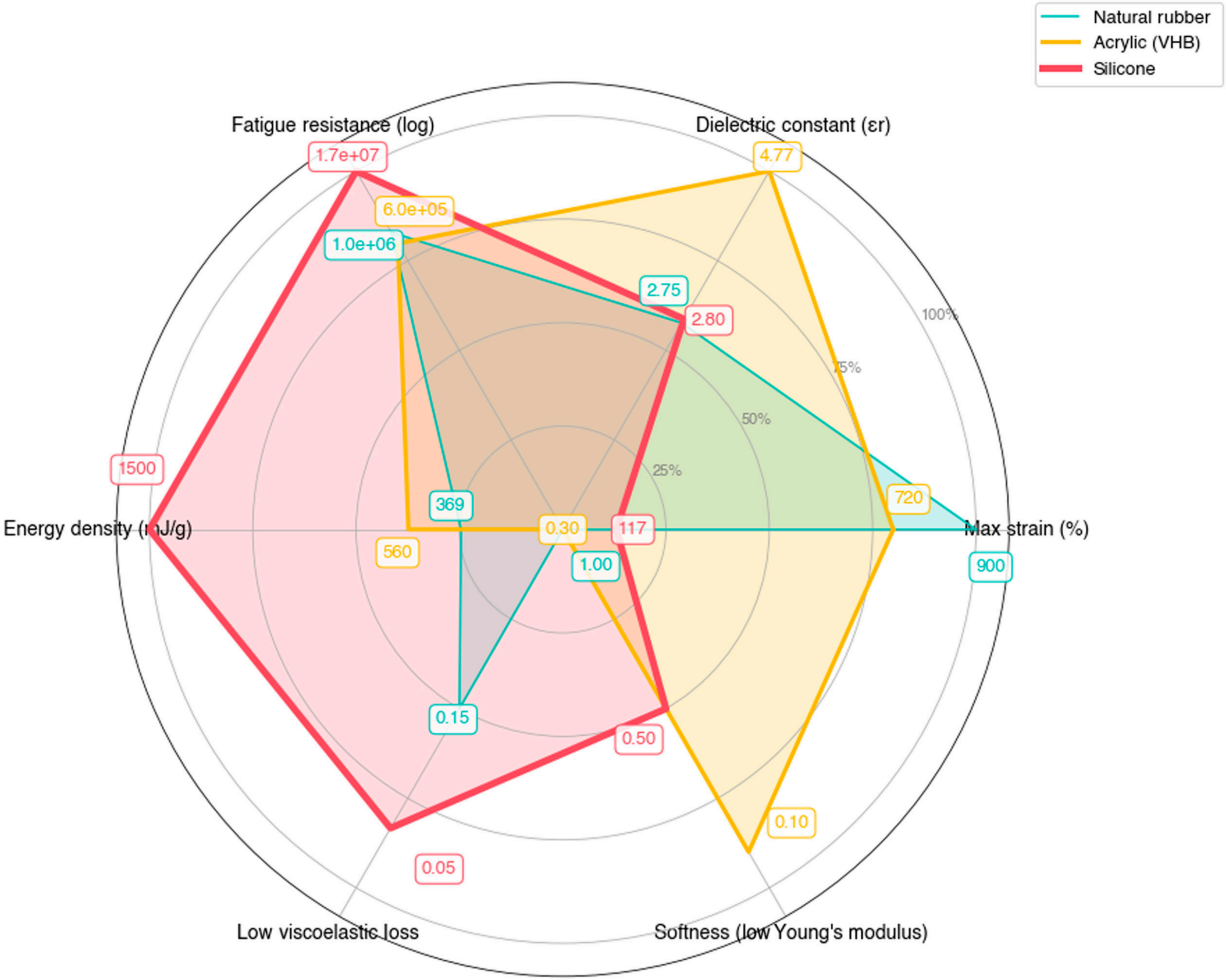
Comparative performance radar chart of key dielectric elastomer classes with normalized critical properties (axes for loss and modulus are inverted).

To visually summarize the complex trade-offs between the major classes of DEs, a performance radar chart is presented in Fig. 2. The materials selected for this comparison—silicone (PDMS), acrylic (VHB), PVDF, a silicone/MWCNT composite, and NR—were chosen to be highly representative of the 3 main categories discussed in this review: silicone-based, nonsilicone, and composite materials, each showcasing distinct performance strengths and weaknesses. This chart highlights the distinct performance profiles of each material, providing an intuitive basis for the detailed comparative analysis that follows. It elucidates the core principle of material selection for DEGs: There is no single “best” material, only the “most suitable” for a given application. For instance, silicone emerges as the “all-rounder” with a balanced shape, its key strength being high fatigue resistance (1.73 × 10^7^ cycles [29], compared to VHB’s 1 × 10^6^ cycles at 20 psi design stress under full reversal loading [30]). In contrast, acrylic (VHB) shows a specialized profile with a pronounced spike in maximum strain, counterbalanced by a significant weakness in fatigue resistance. PVDF also demonstrates a specialized profile, excelling in dielectric constant but performing poorly in terms of strain and mechanical softness. Meanwhile, the composite example illustrates the goal of material engineering: to significantly boost a specific property like the dielectric constant, often at the expense of other metrics such as cost-effectiveness.

The radar chart performance parameters are extracted from peer-reviewed literature. For silicone (PDMS): maximum strain (117%), dielectric constant (2.8), and energy density (1,500 mJ/g) from [31]; fatigue resistance (1.73 × 10^7^ cycles) from [29]. For acrylic (VHB 4910): maximum strain (720%) from [5,32]; dielectric constant (4.77) from [33]; fatigue resistance (1 × 10^6^ cycles at 20 psi stress level) from [30]; energy density (560 mJ/g) from [34]. For NR: maximum strain (900%) from [35]; dielectric constant (2.75) from [36]; fatigue resistance (10^6^ cycles) from [37]; energy density (369 mJ/g) from [38]. The viscoelastic loss parameter reflects the well-documented characteristics of each material class: Silicone exhibits low loss (high efficiency) [31], VHB shows high viscoelastic loss (20% to 40% efficiency) [5], and NR demonstrates intermediate loss behavior (7.2% efficiency) [38]. Young’s modulus values represent the typical stiffness range for each material class, with silicone at 0.5 MPa [31], VHB at 0.1 MPa (characteristic of soft acrylics), and NR at 1.0 MPa (typical for elastomers). All parameters were normalized using linear scaling (value/maximum) for strain, dielectric constant, and energy density; logarithmic scaling for fatigue cycles to accommodate the wide range of values; and inverse scaling (1 − value/maximun) for loss and modulus, where lower values indicate superior performance.

The choice between silicone and nonsilicone materials hinges on application-specific requirements [18]. Silicone elastomers dominate scenarios prioritizing reliability and elasticity, such as wearable sensors or medical devices. Their fatigue life (1.73 × 10^7^ cycles [29]) significantly exceeds that of acrylics (VHB: 1 × 10^6^ cycles [30]) while maintaining thermal stability across a wider temperature range, which, despite enabling >300% strain, suffer from significant viscoelastic losses. PVDF and graphene–epoxy composites, while niche, excel in high-energy-density systems but face scalability and cost barriers.

Silicon-based dielectric materials exhibit distinct advantages in lab-tested fatigue resistance. Under typical test conditions (electric field 70 to 75 MV/m, frequency 1 Hz, pure shear deformation), silicone dielectrics achieve 2 × 10^6^ cycles—200× longer than NR’s 10^4^ cycles under identical loading [5]. More comprehensive testing reports silicone elastomers reaching 8.5 × 10^4^ cycles [39] and up to 1.73 × 10^7^ cycles [29], compared to VHB’s 1 × 10^6^ cycles at 20 psi design stress under full reversal loading [30]. This represents 10× to 2,900× improvement in fatigue life. Their environmental resilience stems from extremely low electrical conductivity (1 to 4 orders of magnitude lower than VHB and rubber) and extended charge retention (self-discharge time constant $\tau_{d}$ = 500 to 50,000 s versus VHB’s 7 to 37 s), enabling reliable operation in low-frequency applications (<1 Hz) such as ocean wave energy harvesting [5], and cost-efficiency ($45 to $60/kg). Their superior elasticity (117% to 450% recoverable strain [5,31]), stable energy conversion efficiency (5% to 40% depending on formulation [40,41]), and resistance to environmental degradation make them ideal for long-term applications like medical implants and marine energy harvesters.

In contrast, nonsilicone alternatives demonstrate exceptional deformation capabilities—VHB and ECOflex achieve 300% to 1,100% strain—albeit with trade-offs in thermal stability and hysteresis loss. Acrylic elastomers (VHB) are widely used in laboratory demonstrations due to their large strain capacity and relatively low modulus of elasticity, making them suitable for large deformations with limited input forces. However, their high viscoelasticity and frequency dependence limit applications in fast and reliable drives. Natural or synthetic rubber offers greater breakdown strength and lower conductivity for specific strain ranges, with the added benefit of eco-friendly properties including low carbon footprint and biodegradability. PVDF provides unique piezoelectric properties (20 to 30 pC/N) for precision sensing, while graphene–epoxy nanocomposites offer enhanced EMI shielding (60 to 80 dB) at premium costs ($800 to $1,200/kg).

The selection matrix reveals fundamental trade-offs (Tables 4 to 6). Silicon materials provide 2× to 3× longer service life than acrylic elastomers but limit maximum strain to 200%. Silicone elastomers demonstrate low mechanical losses and very low conductivity, making them efficient for operation in the low-frequency range, while offering superior design flexibility due to their ability to be fabricated in any desired thickness and shape. Nonsilicone options like ECOflex enable extreme deformations (1,100%) at reduced costs ($80 to $100/kg), yet require frequent replacement in high-cycle applications. Styrene rubber and NR are particularly suitable for large strain actuators and generators, especially where large elastic stiffness is not required, while acrylic (VHB) elastomers are primarily used in demonstrators and laboratory prototypes due to reliability and lifetime limitations.

**Table 4.** Performance comparison of DEG materials

Material	Dielectric constant	Max strain (%)	Applications
*Silicon-based*			
Silicone dielectric	2.8 [31]	117 [31]	Flexible actuators, high-frequency applications
Silicone elastomer	2.85 [5]	450 [5]	Soft robotics, high-voltage actuators
*Nonsilicone*			
VHB 4910	4.77 [33]	600–720 [5,32]	Wearable sensors, marine energy
Natural rubber	2.75 [36]	900 [35]	Motion sensing, biomedical
PVDF	12.76 [146]	6.9 [147]	High-voltage actuators, energy harvesting
Graphene–epoxy	8.23 [148]	5.32 [149]	Structural composites, energy harvesting
ECOFLEX 00-30	2.8 [150]	800 [151]	Flexible electronics, soft actuators

**Table 5.** Comparative advantages of dielectric materials for DEG applications

Category	Silicon-based materials	Nonsilicone materials
Performance metrics	Low viscoelastic loss (2–6× lower than VHB), fast response time, extremely low conductivity (1–4 orders of magnitude lower than alternatives), fatigue resistance (up to 1.73 × 10^7^ cycles) [29], extended charge retention ($\tau_{d}$ = 500–50,000 s) enabling low-frequency operation [5]	Higher strain capacity (VHB, natural rubber), higher dielectric constant (PVDF, graphene–epoxy), eco-friendly properties (natural rubber), superior electromechanical coupling (VHB)
Application suitability	Long-term reliability applications, marine environments, high-frequency operations, design-critical implementations, industrial-scale deployments	Laboratory demonstrations (VHB), large-strain applications (ECOFLEX), low-frequency energy scavenging (natural rubber), specialized sensing (PVDF), EMI shielding (graphene–epoxy)
Primary applications	Marine environments, high-frequency operations, industrial-scale deployments	Motion sensing, energy harvesting, wearable electronics, flexible actuators
Limitations	Limited strain capacity (<220%), lower dielectric constant, moderate energy density	High viscoelasticity (VHB), limited cycle life, frequency dependence, higher production costs, moisture sensitivity

**Table 6.** Comprehensive summary table (I) of unified indicators (dielectric constant, maximum strain, energy efficiency, energy density, and fatigue cycles)

Material	Dielectric constant	Maximum strain (%)	Energy efficiency (%)	Energy density (mJ·g^−1^)	Fatigue cycles
Fluorinated silicone	7.37 [152]	138–205 [153]	N/A	N/A	N/A
TiO_2_/silicone foam	5.06 [154]	330 [154]	8.84 [6]	64 [155]	42,000 [155]
Dipole-modified silicone	21 [156]	9.97 [157]	N/A	N/A	N/A
MWCNT/PDMS composite	10 [158]	145 [159]	3.02 [160]	0.55 [160]	15,000 [161]
NS-CNTs/silicone	3.2–8.6 [162]	100–250 [163]	N/A	N/A	N/A
UNDE (BNNS)	1.78 (100 kHz) 1.16 (1 MHz) [164]	8–10 [144]	N/A	N/A	N/A
CNT–Al_2_O_3_	10–300 [165]	81 [166]	N/A	N/A	N/A
ZrO_2_–PDMS	4–6 [167]	78 [168]	N/A	350–1,000 [167]	10^3^−10^5^ [119]
Graphene–PU	9–10 [169]	605–2,170 [170]	N/A	1,700 [171]	N/A

Emerging hybrid approaches combine silicon’s durability with PVDF’s piezoelectric response [42], demonstrating 40% energy density improvements in prototype self-powered sensors. Future research directions should focus on optimizing elastomer composition and structure [43] to achieve an ideal balance of electrical properties, mechanical performance, and long-term reliability for specific DEG applications.

It should also prioritize hybrid solutions. Combining silicone’s elasticity with PVDF’s piezoelectricity could yield materials tailored for self-powered sensors. Similarly, integrating NR’s biodegradability with silicone’s durability might address sustainability challenges in marine applications. Advances in nanofiller dispersion and interfacial engineering will be critical to mitigating leakage currents and enhancing breakdown strength in composite systems (Table 7).

**Table 7.** Comprehensive summary table (II) of unified indicators (dielectric constant, maximum strain, energy efficiency, energy density, and fatigue cycles)

Material	Dielectric constant	Maximum strain (%)	Energy efficiency (%)	Energy density (mJ·g^−1^)	Fatigue cycles
VHB elastomer (4910)	4.77 [33]	600–720 [5,32]	20–40 [5]	560 [34]	1 × 10^6^ (at 20 psi stress) [30]
PVDF	12.76 [146]	6.9 [147]	13.5 [172]	1.97 × 10^4^ [173]	1 × 10^7^ [174]
PUA	6.35 [175]	590–600 [145]	1.56 [176]	1.37 [177]	1,050 [178]
Natural rubber	2.75 [36]	900 [35]	7.2 [38]	369 [38]	10^5^−10^6^ [37]
PEEK	3.1–3.9 [179,180]	43.1 [181]	97 [182]	2,900 [182]	10^4^−10^6^ [183]
Silicone dielectric	2.8 [31]	117 [31]	40.5 [41]	100–1,500 [31]	1.73 × 10^7^ [29]
Silicone elastomer	2.85 [5]	450 [5]	5.01 [40]	0.71 [40]	8.5 × 10^4^ [39]
Graphene–epoxy	8.23 [148]	5.32 [149]	N/A	N/A	10^5^−10^6^ [184]
ECOFLEX 00-30	2.8 [150]	800 [151]	N/A	N/A	N/A

## Dielectric Elastomer Generators

### Working principles

DEG operates based on dielectric elastomer transducer (DET) principles. DET has excellent flexibility and a simple structure. It is lightweight and low-cost and can accommodate large mechanical strain. It has broad application prospects and has attracted widespread attention. A typical DET device consists of a DE film sandwiched between 2 flexible electrodes [44]. When DET operates in generator mode, it is called a DEG. DEG is an electrostatic generator composed of elastic insulating polymers with stretchable and variable capacitance properties, which can convert mechanical energy into electrical energy during stretching and release. Its working principle is based on the electrocontraction effect [45]. It has excellent energy conversion efficiency, is lightweight and flexible, and can adapt to various shapes and sizes [46], providing a simple and effective way to harvest energy from natural movements such as waves, tides, and human body movements. DEG has a sandwich-like structure, with flexible electrodes coated on both sides of the DE film to form a stretchable flexible capacitor. The DE film deforms under the action of external force, causing a change in capacitance. When the film recovers from the stretched state to the released state, DEG converts the mechanical energy generated by the polymer deformation into electrical energy. Its energy collection process is based on the electromechanical properties of the flexible DE. By periodically applying and withdrawing the electric field, the elastomer vibrates. With the help of the connection between the mechanical structure and the generator, energy collection and conversion are achieved.

Electrocontractile grafted elastomers are composed of 2 polymers, the first of which has a flexible skeleton and the other of which is grafted onto the first, presenting a structure with crystalline regions. The crystalline region is both a physical crosslinking point and a polarized part, which determines how the material deforms under the action of an electric field. DEG uses flexible, highly elastic materials with a Young’s modulus between 1 and 5 MPa, such as VHB 4910, which can achieve a tensile strain greater than 100%. This type of flexible, highly elastomeric material is easy to process and has excellent energy conversion efficiency and dynamic response characteristics. It generates electrical deformation under the action of an electric field and converts periodic vibrations into electrical energy with the help of a mechanical structure.

Figure 1B shows the topological structure of DEGs. The simplest DEG planar topology has deformation kinematics in which stretching is caused by a load applied to the electrode periphery. This is known as equibiaxial [8] stretching [Fig. 1B (i)] and pure shear stretching [Fig. 1B (ii)], also known as strip-biaxial extension [47].

Equibiaxial stretching refers to the uniform stretching of the DE film in all directions within the electrode plane, achieving the maximum capacitance change at the maximum tensile strength allowed by the material [48]. Deformation can be achieved by a peripheral wire-drawing device [Fig. 1B (i)] [34] or by applying vertical compression [49], the latter of which can build a self-supporting structure without a rigid frame. Pure shear stretching is achieved by applying forces at both ends of the strip to maintain a constant transverse stretch, thereby producing longitudinal expansion [Fig. 1B (ii)] [50]. Pre-stretching is usually applied to avoid tension loss. Although the capacitance change is limited, the structure is simple and easy to implement.

To overcome the limitations of equibiaxial and pure shear DEGs, the edges of the DE membrane are connected to a closed-chain structure to achieve a planar DEG. The diamond DEG shown in [Fig. 1B (iii)] [51] consists of a diamond-shaped DEG membrane. A 4-bar linkage is used to clamp the diamond membrane at its periphery. The membrane is pre-stretched biaxially along the diagonal lines, and the capacitance area is maximized when it is square. Due to the kinematics of the mechanism, the increased diagonal stretching leads to relaxation in the vertical direction, so the area strain achieved by this structure is more limited than that of equibiaxial or pure shear modes.

The conical DEG [Fig. 1B (iv)] [52] consists of a uniformly pre-stretched annular membrane, with the outer edge fixed to the annular frame and the inner edge connected to a rigid disk. The membrane is deformed into a cone by applying a longitudinal force to the disk. The capacitance is minimum when the membrane is flat and increases with the displacement of the disk. The structure produces the main stretch in the meridian direction and is basically unchanged in the circumferential direction. The kinematics are similar to pure shear. Unlike conical actuators that require axial preload [53], conical DEGs do not require a biasing mechanism and can fully utilize their deformation range. Two oppositely preloaded conical units form an active-antagonistic structure by sharing a central disk, thereby achieving bidirectional power generation [54].

The circular diaphragm-type pneumatic DEG [Fig. 1B (v)] can utilize the periodic pressure changes in the fluid. The structure consists of a circular DEG film that is uniformly pre-stretched in a flat surface. The capacitance increases when the pressure drives the film to expand into a bubble shape. This topology has been widely studied due to its simple structure and the large area strain that can be achieved over most of the electrode surface, which is close to equibiaxial [38].

During electrical excitation and energy harvesting, material parameters such as dielectric constant, strain strength, EB strength, and volume conductivity critically influence the system’s capacitance, bias voltage, and charge loss, thereby determining the energy generated in a single cycle [48]. The external environment and the nature of the energy itself are factors that affect energy conversion [8,52]. Young’s modulus, elongation at break, and mechanical loss are the mechanical properties of DEs. Dielectric constant, EB strength, and volume conductivity are electrical related properties. Stretching mode and circuit design are device variables, and stretching ratio and bias voltage are operating variables, which are external environmental factors. The inherent properties of the material determine the maximum range of the operating variables. The elongation at break and the EB strength of DE materials limit the maximum stretching multiple and the maximum bias that can be applied. Therefore, by studying DE materials with different elongations at break and breakdown field strengths, the energy harvesting performance under different stretching multiples and bias conditions can be compared equivalently.

The energy harvesting mechanism is theoretically scale-independent and resonance-independent, and can be directly coupled to linear motion [55]. It can operate in a nonresonant state and produce high energy density [34], making DEG suitable for low-complexity, miniature energy harvesters. It efficiently harvests energy from a variety of frequencies, including extremely low-frequency motion such as the swaying of tree branches [56] and the rise and fall of waves [57]. DEG can collect energy from daily human motions by virtue of its softness [49], which refers to its high flexibility and mechanical compliance that enable close contact with the human body and tolerance to continuous deformation. This energy conversion mechanism does not rely on resonance and can operate in a nonresonant state.

The energy harvesting cycle of DEG consists of several stages:

Stage 1: Mechanical energy is used to stretch the generator to increase its capacitance.
Stage 2: When the capacitance reaches its maximum value, a charge is applied to the generator.
Stage 3: The generator begins to mechanically contract, and the capacitance decreases. Opposite charges are pulled apart, and like charges are compressed, which creates a stronger electric field to increase the electrical energy of the charge.
Stage 4: The charge is extracted, and energy conversion is achieved by adopting a high-energy state [49].

In the energy cycle, Eq. (1) converts the associated energy generated by the cycle at a constant charge *Q* into electrical energy.

$$E_{\mathrm{pro}}=\frac{1}{2}\left(C_{\min}V_{\max}^{2}-C_{\max}V_{\min}^{2}\right)$$ (1)

where $C_{\min}$ and $C_{\max}$ are the minimum and maximum capacitances, respectively, in the relaxed membrane and polarized membrane in the cycle. $V_{\max}$ and $V_{\min}$ are the maximum and minimum voltages in the same state, respectively. The charging and discharging processes apply to the 2 extreme states of maximum and minimum capacitance. When such a structure switches from a high capacitance value to a low capacitance value, an energy conversion mechanism occurs. The classical cycle requires initial polarization energy, such as constant charge *Q*, constant voltage *V*, and constant electric field *E*, and the boost converter is part of the generator circuit [45].

There are 3 energy harvesting modes in one cycle of DEG: constant charge *Q*, constant voltage *V*, and constant electric field strength *E*. Constant charge *Q* obtains more energy in one cycle, which is common in DEG energy harvesting applications. The energy conversion process of DEG has 4 steps, as shown in Fig. 1B.

In the first step, DEG possesses the minimum capacitance ($C_{\min}$) in the initial state. Upon application of an external mechanical stretching force, the DEG undergoes in-plane expansion and thickness reduction, resulting in a capacitance increase to $C_{\max}$. This mechanical deformation provides the necessary mechanical energy for subsequent electrical energy conversion. The capacitance calculation formula is as follows:

$$C=\frac{\varepsilon_{r}\varepsilon_{0}S}{D}$$ (2)

where $\varepsilon_{r}$ and $\varepsilon_{0}$ are the relative and absolute dielectric constants (8.85 × 10^−12^ F·m^−1^), respectively. *S* and *D* are the area and thickness of the DEG, respectively.

In the second step, while maintaining the stretched configuration ($C_{\max}$), a bias voltage is applied across the electrodes, injecting an equal amount of opposite charges +*Q* and −*Q*. This establishes an electric field within the dielectric layer and stores electrical energy in the form of electrostatic potential. After charging, the power supply is disconnected, and the amount of charge in DEG remains unchanged.

In the third step, the mechanical force is removed to relax the DEG to a state close to the original state. This relaxation leads to an increase in thickness and a decrease in electrode area, causing the capacitance to drop from $C_{\max}$ to $C_{\min}$. As the charge remains constant, the voltage rises, the electric field strengthens, and the stored electrical energy increases. The energy generated is calculated as follows:

$$\Delta E=E_{2}-E_{1}=\frac{1}{2}\left(C_{2}V_{2}^{2}-C_{1}V_{1}^{2}\right)$$ (3)

**Fig. 3.**
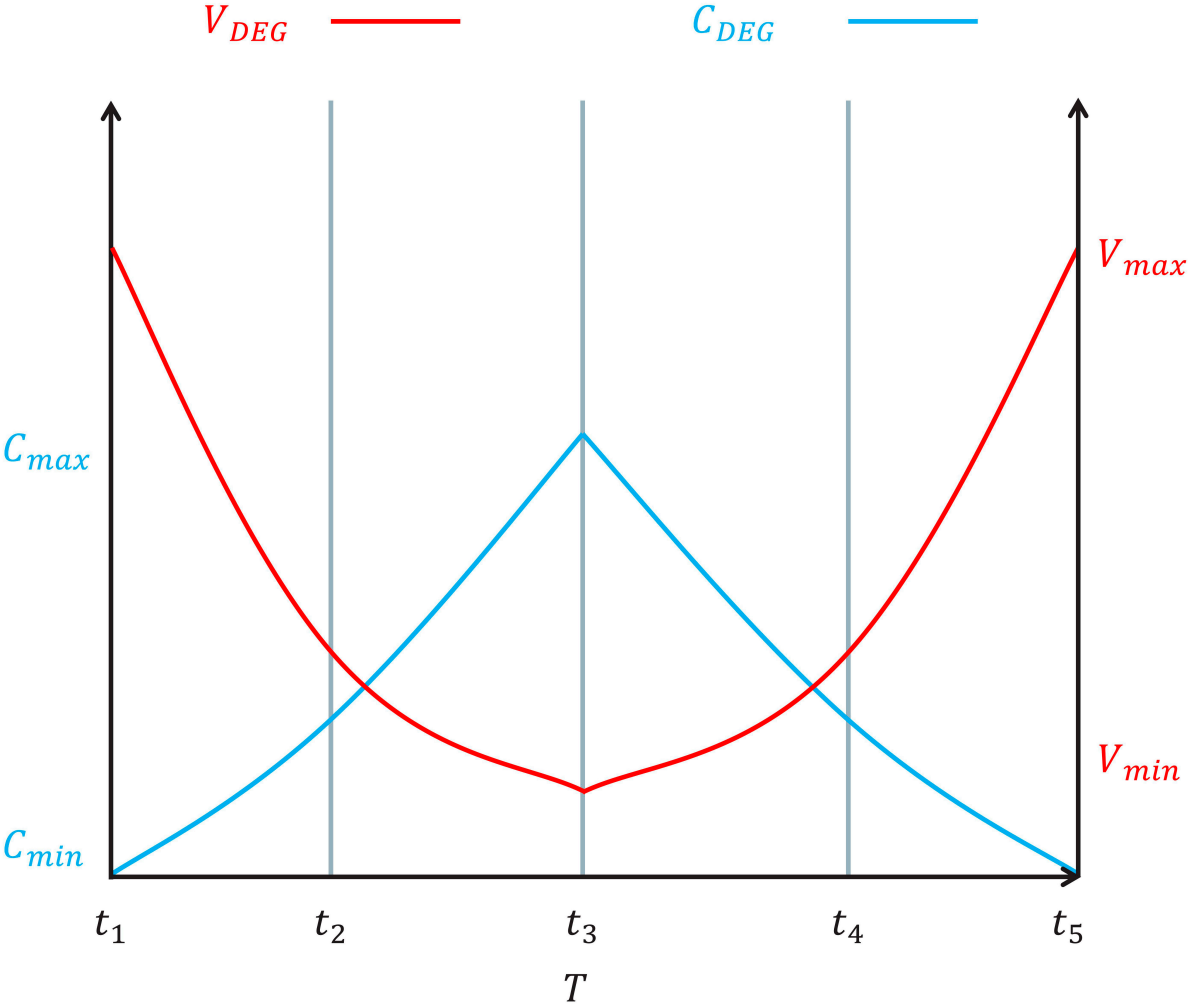
Waveforms of a DEG over one operating cycle in the capacitance–voltage plane, based on the concepts described in [43].

The amount of charge in DEG remains unchanged. According to the equation $Q=\left(C_{2}V_{2}^{2}-C_{1}V_{1}^{2}\right)$, the capacitance and voltage of DEG changing in one cycle are shown in Fig. 3 [43]. Equation (3) is rewritten as:

$$\Delta E=\frac{1}{2}\left(C_{2}V_{2}^{2}-C_{1}V_{1}^{2}\right)=\frac{1}{2}C_{1}V_{1}^{2}\left(\frac{C_{1}}{C_{2}}-1\right)$$ (4)

where $S_{1}$, $d_{1}$, and $V_{1}$ are the area, thickness, and bias voltage, respectively, of DEG in state III, and $S_{2}$ and $d_{2}$ are the area and thickness, respectively, of DEG in state IV. When DEG is subjected to force or released, the volume of DEG is assumed to remain unchanged. $V=S_{1}\times d_{1}=S_{2}\times d_{2}$. According to Eq. (2), Eq. (4) can be rewritten as:

$$\Delta E=\frac{1}{2}\frac{\varepsilon_{r}\varepsilon_{0}S_{1}}{d_{1}}V_{1}^{2}\left(\frac{S_{1}}{S_{2}}\times \frac{d_{1}}{d_{2}}-1\right)=\frac{1}{2}\frac{\varepsilon_{r}\varepsilon_{0}S_{1}}{d_{1}}V_{1}^{2}\left(\frac{S_{1}^{2}}{S_{2}^{2}}-1\right)$$ (5)

The fourth step is the discharge step. When the capacitance reaches the minimum value ($C_{\min}$), the output circuit is closed, and the stored charges are discharged through the external load, delivering electrical power. The DEG thereby returns to its initial state, completing a full energy conversion cycle.

### Performance comparison and recent improvements

The energy output performance of DEG in a single cycle is measured by 2 key parameters: energy density ($E_{\text{density}}$) and electromechanical conversion efficiency (η). $E_{\text{density}}$ is the ratio of the generated energy to the material mass (*m*), and η is the ratio of the generated energy to $W_{\text{mech}}$. $W_{\text{mech}}$ is the mechanical energy input in one cycle, which is calculated from the force–displacement integral curve of DEG shown in Fig. 1B (a). $E_{\text{density}}$, $W_{\text{mech}}$, and η are calculated as follows:

$$E_{\text{density}}=\frac{\Delta E}{m}$$ (6)

$$W_{\text{mech}}=\int_{S_{0}}^{S_{1}}\left(F_{\text{stretch}}-F_{\text{release}}\right)\;\mathrm{dS}$$ (7)

$$\eta =\frac{\Delta E}{W_{\text{mech}}}$$ (8)

In recent years, researchers have made progress in improving the energy density and electromechanical conversion efficiency of DEG. For example, Fan and Chen [58] significantly enhanced the output performance of DEG by reducing the pre-stretching ratio and extending the cycle period. It was discovered by Zhou et al. [59] that avoiding tension loss can successfully improve energy conversion efficiency. A stretchable circuit was integrated into the membrane material, and an energy density of 10 mJ/g at a conversion efficiency of 12% was achieved by McKay et al. [60]. In a laboratory setting, an energy density of 18.9 mJ/g and an energy conversion efficiency of 18.3% were successfully attained by Wang et al. [61] by combining the charging and stretching modes. Using an equibiaxial mechanical loading setup on a small-scale prototype, Huang et al. [34] attained an energy density of 560 mJ/g and an energy conversion efficiency of 27%. By optimizing the triangular design, a subsequent lab-based study reported a maximum energy density of 780 mJ/g [62]. In terms of low-frequency energy conversion, the energy conversion efficiency of DEG has exceeded 100 mJ/g, which is at least one order of magnitude higher than that of traditional piezoelectric and electromagnetic generators [48,63]. Figure 1B (b) shows the operation of DEG under 4 failure mode conditions [48,64]: EB, EMI, material rupture (MR), and strain loss (LT). Under the same conditions, the generation of electrical energy depends mainly on the degree of capacitance change of dielectric elastomer capacitor (DEC) during electromechanical cycling [48]. DEG is stretched by applying load conditions close to the electrical and mechanical limits of the elastomer to maximize the capacitance change [34]. By optimizing the material properties and stretching method, the energy density of DEG can reach the order of 50 to 1,000 mJ/g [50,61]. Jiang et al. [52] combined equibiaxial pre-stretching with conical stretching while adjusting the input bias voltage pre-stretching ratio, attaining an energy density of 130 mJ/g and a conversion efficiency of up to 40%. Despite the significant improvement in experimentally achieved energy density, DEG is still lower than the predicted theoretical maximum energy density of 1.7 J/g [8], which is due to the viscoelastic effect and leakage current of DEG [65]. In addition, the energy output of DEG is affected by factors such as dielectric strength and temperature [66], and the deformation mode of DEG also has a significant impact on energy collection. Therefore, material selection and deformation mode are the key to improving output performance.

DE materials with low elastic modulus and high dielectric constant directly affect the energy collection and output performance of DEG, especially in terms of breakdown dielectric strength, dielectric properties, and mechanical properties. In addition, the structure of DEG is closely related to its deformation mode, and the structural design directly affects energy collection. Based on prototypes that harvest energy from a variety of mechanical sources (including human motion, wind energy, and wave energy), the key factors related to mechanical energy harvesting, material properties, deformation mode, and prototype design are analyzed, covering small scale (such as wind energy and human motion energy) and large scale (such as wave energy) [43]. The output performance of DEG is highly dependent on the properties of the material, and commonly used DE materials are usually stretchable rubber or silicone rubber. Acrylate, rubber, and silicone elastomers are considered to be promising materials for DEG, and their elastic modulus and elongation at break have an important influence on their properties. The large deformation ability of the material significantly changes the capacitance of the DEG to improve the energy output. The breakdown dielectric strength and relative dielectric constant affect the maximum operating voltage and energy density of the DEG, while the energy loss is related to the conductivity and hysteresis loss of the material. The study showed that chemical modification of PMVS with *n*-dimethyl-3-mercaptopropionamide resulted in a DE with excellent properties. Tension was applied by pre-stretching the material to improve the performance of DEG. Pre-stretching plays an important role in acrylic elastomers by changing the geometry of the actuator to improve breakdown strength, efficiency, and actuation strain. Unlike rigid frames, the use of nylon fiber reinforcements can maintain the pre-stretched state of the elastomer, allowing the actuator to move in one direction. When an electric field is applied, the actuator of DEG changes its shape in response to the applied electric field, resulting in a dielectric polarization effect in the DE, that is, the transfer of positive and negative charges to each other. This deformation includes stretching, squeezing, or twisting and ultimately releases mechanical energy. DEG is more dependent on the strain value at fracture, so a large tensile deformation needs to be applied to it when constructing a stretch sensor. When the electric field is applied, buckling and wrinkling occur when the mechanical stress on the film changes from tension to compression, resulting in out-of-plane deformation. DEG seeks high dielectric constant elastomers that convert electrical energy into mechanical energy while also converting thermal energy into electrical energy and vice versa. The released mechanical energy can be converted into electrical energy through mechanical connection to equipment such as generators.

The elastomer of DEG fatigues after multiple stretching and contraction cycles, affecting its long-term service life, and therefore needs to be replaced regularly. Under controlled, accelerated laboratory testing, silicon-based DE actuators have demonstrated a service life of up to 10^8^ cycles. The fatigue life of DE materials is affected by many factors, including durability under electric fields and the electrical connectivity of electrodes during large strain cycles. Their life performance under mechanical stretching (no voltage) and cyclic charge and discharge (fixed stretch state) was studied. The results show that, based on material-level testing, the mechanical fatigue life of NR and synthetic rubber is 10^6^ to 10^8^ cycles, and their use can improve the long-term stability of DEG. The usability of DEG depends on its excellent dielectric and mechanical properties, and is also affected by commercial availability [5].

During the vibration cycle, deformation of the elastomer leads to material fatigue. The significant hysteresis effect caused by high energy dissipation requires high electric field strength, and electrode fatigue under large strain is also a potential problem [67]. The elastomer of DEG is ductile and can adapt to different shapes and sizes. However, the upper limit of DEG performance is limited by mechanical failures such as EB threshold, tension loss, and maximum allowable longitudinal stretching. Two representative cases are numerically analyzed based on the maximum allowable stretching of the cycle: one is a relatively rigid DE with a small reversible stretching range; the other is an elastomer with greater ductility that can be stretched to several times the reference length [67].

DEGs are made of cheap rubber or elastic polymers and are cheaper to manufacture than other energy generators. Their deformable parts are made using low-cost materials and processes and are lightweight and compatible with sensors, making the actuators inexpensive [45,67]. The corrosion resistance of DEGs depends on the selected materials, and specially treated elastomers can improve their corrosion resistance in seawater. Compared with traditional PTO systems, DEGs are lower in cost, simpler in structure, and more corrosion resistant [5,43].

However, DEG is particularly sensitive to environmental conditions, especially drastic changes in temperature and humidity, which can affect the mechanical properties of the elastomer [44], so additional control and regulation are required to ensure stable performance. DEG may not be strong enough under extreme operating conditions, so tougher film materials have been developed for DE applications, such as the tougher commercial NR elastomer [5].

DEG has certain energy losses in the process of converting mechanical energy into electrical energy. Although the high expansibility and good force coupling of elastomers make the conversion efficiency of elastomer motors relatively high, the overall efficiency of DEG is still constrained by the efficiency of the electronic devices it drives. The mechanical loss of DEG mainly comes from rate-dependent viscosity loss, which increases with the increase of strain rate. Although some DE materials have rate-independent hysteretic pseudoelastic properties, if the mechanical energy dissipated and stored in the DE material is high, the mechanical loss will cause a significant decrease in the efficiency of DEG, although this does not inhibit its ability to output positive power or affect the maximum convertible energy density. The electrical loss is caused by the R–C dynamic effect inside the DEG, including the leakage current of the dielectric layer caused by the finite resistivity of the DE material and the uneven voltage on the electrode caused by the finite conductivity of the flexible electrode material [5]. These factors work together to reduce the efficiency and convertible energy density of DEG.

The energy conversion efficiency of DEG is affected by mechanical and electrical losses. The core task of the regulation unit is to synchronously control the periodic motion of the DEG transducer to pump the charge flow. When the deformed DEG pumps the charge to the output, the analog electrical signal generated depends on the mechanical deformation of the DEG [68]. However, the generated electrical signal must be processed to produce a usable output signal. The circuits are used in the laboratory for energy conversion, and the nonlinear switching of capacitors as well as the material dynamics and viscoelasticity of the materials lead to a certain complexity [69]. In addition, DEG requires higher bias voltage (500 to 2,000 V) to activate, which makes the integration of interface circuits difficult [70].

Researchers have proposed a variety of electronic circuit designs for DEG interfaces, and a large number of complex interface circuit schemes have been reported. Pelrine et al. [31] proposed a voltage divider circuit to achieve the measurement of the display unit by reducing the output voltage.

Advancement of electronics related to DEG has been summarized in [6]. A Buck/boost converter using 2 STP3N150 (1,500 V) metal–oxide–semiconductor field-effect transistors (MOSFETs) was proposed by Due et al. [71]. The study provides a way to use DE as a transducer for harvesting mechanical energy at a lower frequency of vibration of DE. A prototype circuit for DEG under high-frequency vibration input (relatively small displacement of DE sample) was designed by He et al. [72]. The prototype was simulated using PSPICE and constitutes a low-pass filter followed by a switch-mode converter. A forward path circuit for DEG with a distinct pattern of vibration was developed by [73]. The electrical output of DEG was processed through regulation and switch-mode conversion stages, with the circuit operated in an open-loop condition. A circuit prototype for a dielectric polymer energy harvesting system was proposed by Ge et al. [74]. The study reveals a prototype that employs a transformer for boost and buck purposes, simulated in MATLAB. A multilevel high-voltage converter for driving DEGs was introduced by Graf et al. [75], using a multilevel boost converter to bias the DEG. Frequency-domain tradeo-ffs for DEGs were studied by Zanini et al. [76]. The frequency-domain behavior of DEGs undergoing 2 different cycles and different biasing voltages was compared. An electrical model for a DEG was proposed by Panigrahi and Mishra [68]. The proposed model and experimental investigations reveal a gain of 2.54 at a DEG operating frequency of 2 Hz, using a fly-back converter to step down the voltage. An autonomous electrostatic energy harvester with voltage boosting was designed by Illenberger et al. [77]. A self-priming circuit was used for voltage boosting in DEG, and a potential divider to measure the output. An energy conversion unit using donut-shaped DEG with relative analysis of stretch dependence capacitances was developed by Sadangi et al. [78]. A controlled conditioning interfacing unit was used with MOSFET.

### Theoretical models: Limitations and advanced simulation

Traditional electromechanical models can describe the energy conversion process of wave energy devices, such as DEGs. However, they often treat waves as external excitations, neglecting the strong coupling between waves and structures, making it difficult to accurately reflect dynamic behavior under complex operating conditions [79]. Recent advances in computational fluid dynamics (CFD) technology have enabled the capture of nonlinear interactions between waves and floating structures at higher resolutions, improving predictions of device motion, loads, and hydrodynamic efficiency. Building on this foundation, fluid–structure interaction (FSI) methods have been gradually introduced to study the motion and fatigue characteristics of floating wave energy devices under extreme sea conditions, revealing the importance of coupling effects in survivability prediction [80]. A further trend is to integrate electrical or PTO models into the CFD–FSI framework. Through cross-scale joint simulations, device-level damping or stiffness characteristics can be linked to system-level energy capture efficiency, motion response, and load distribution. This allows for a more realistic understanding of the performance and survivability of wave energy devices under extreme sea conditions [81]. A modular floating platform–WEC joint model can reveal the complex interactions between hydrodynamic properties and PTO parameters. Mathematical modeling studies have shown that for floating WECs, mooring not only determines the device’s stationary position but also forms dynamic feedback with the device’s motion through nonlinear damping and restoring forces. Therefore, in coupled modeling, the interaction between the PTO, platform, and mooring cannot be ignored; otherwise, it is difficult to accurately capture the system’s dynamic behavior [82]. In practice, such co-simulations are commonly implemented using open-source frameworks such as OpenFOAM for CFD coupled with CalculiX for structural analysis via the preCICE coupling library, or DualSPHysics combined with Project Chrono for smoothed particle hydrodynamics (SPH)-based wave–structure interaction. Commercial alternatives include Siemens Simcenter STAR-CCM+ and ANSYS Fluent with integrated FSI capabilities. For wave energy applications, specialized toolchains are often built on top of these general-purpose solvers. Reynolds-averaged Navier–Stokes (RANS)-based numerical wave tanks that use wave-generation toolboxes such as waves2Foam are widely employed to study WEC hydrodynamics and survivability under steep and breaking waves [83]. At the system level, wave-to-wire frameworks based on industry codes such as OrcaFlex link hydrodynamics, mooring, PTO, and control models in a single simulation environment and are now commonly used in WEC design and optimization studies [84].

With the application of multiphysics modeling methods within the CFD–FSI framework, researchers are able to more systematically capture the nonlinear dynamic response of PTOs under complex sea conditions. This not only provides a more realistic numerical environment for control strategy optimization but also opens up new paths for structural safety and reliability assessment [83]. Recent research has demonstrated that relying on a single electromechanical model fails to accurately capture the extreme loads and failure mechanisms of wave energy devices under extreme sea conditions. However, combined multiphysics modeling combining fluid–structure–PTOs enables a more comprehensive assessment of device survivability and safety margins, supporting rational design load identification and risk mitigation analysis [85]. In particular, cross-scale simulation methods based on CFD–FSI–electrical coupling enable researchers to directly link the fatigue life and damping properties of materials such as flexible DEs with system-level energy capture efficiency and reliability, enabling more comprehensive performance and life assessment [86]. Supported by a high-fidelity CFD–FSI framework, multiscale coupled simulations not only enable more realistic predictions of the device’s energy capture performance and extreme load distribution but also provide critical feedback for controller design. Combined with recently proposed optimization methods based on predictive control [87], control strategies can effectively mitigate fatigue damage and limit extreme loads while improving energy capture efficiency. Therefore, system-level design for engineering applications must comprehensively consider the coupling effects between hydrodynamic, structural, and electrical subsystems to improve energy conversion efficiency while ensuring the device’s structural safety and long-term reliability in extreme sea conditions [88]. Numerical studies have demonstrated that the use of combined float–mooring–PTO modeling can more accurately predict the energy capture efficiency of flexible wave energy devices and improve estimates of extreme loads and fatigue life, laying the foundation for the engineering application of flexible wave energy technology [89]. Overall, recent developments in CFD–FSI–electrical co-simulation bridge the gap between material-level behavior (fatigue and damping of DEs or hydraulic PTOs) and system-level performance, thus addressing the limitations of purely electromechanical models [90].

### Applications in wave energy conversion

Keplinger et al. [91] studied the deformation phenomenon of DEs near the needle of a corona discharge device, showing that the electric field generated by the needle induces an electric field in the elastic body to cause the material to deform. If there is no direct electrical connection between the electret and the elastomer, by adjusting the electret close to or away from the DE, the electric field is generated or disappeared, thus completing the polarization process to achieve the classic energy cycle. Additionally, power management can be achieved through diodes. The working principle of the scavenger is similar to that of a classic electret generator, where a change in load causes a change in capacitance to rearrange the charge between 2 electrodes. The electrostatic generator uses a resonant structure, harvesting energy from vibrations through changes in capacitance, and is polarized and cycled in the same manner as a DEG. To avoid the use of external supply voltage, transducers that have been polarized by electrets were developed to simplify the design and improve the performance. The DEG interacts with the WEC dynamics through its elastic response. The design of WECs should achieve resonance of the system’s natural frequency with the typical ocean wave frequency, which is key to achieving efficient energy capture from waves. In a DEG-based WEC, the natural frequency is the result of the balance between the inertial and hydrostatic loads and the elastic loads of the DEG [92]. Two different paradigms of DEG-based wave energy concepts have been proposed: point generation and distributed generation [93]. These paradigms are divided into several categories [88] based on their comparison with the ocean wavelength (of the order of 10^2^ m): (a) point absorbers (much smaller than the wavelength); (b) attenuators (with a length comparable to the wavelength) [5].

Point absorber-type DEG WEC is the most widely studied type of system [94,95], based on an existing WEC topology but replacing the conventional generator with a DEG PTO. The earliest point absorber-based DE WEC was proposed by the Stanford Research Institute in the United States and the Hyper Drive Corporation in Japan [96] and tested at sea in 2005. In one of the pioneering field trials for this technology, they built a small buoy WEC demonstrator with a DEG PTO and tested it in mild sea conditions, showing an average output power of 0.25 W and a peak power of 1.2 W. At a higher starting voltage, the system power can reach 11 W [96], and other similar or smaller prototypes have been tested at sea [94] and achieved cycle energy densities of over 100 mJ g^−1^ under dynamic operating conditions [5,94].

Based on the initial success of the early offshore tests of the point absorber distributed wave energy power generation system, researchers further optimized and improved the design of the distributed DEG wave energy power generation system. By adopting innovative structures such as multi-layer distributed power generation stacks and planar single-axis distributed generators, the energy conversion efficiency of the system has been significantly improved. Distributed generators are driven by buoys or oscillating water column (OWC) devices, and convert energy through interaction with waves. The OWC system based on distributed generators has gradually become a research point of great interest due to its simple structure, and its performance has been verified through dynamic modeling, scaling rule formulation, and wave tank tests. For example, the OWC prototype using VHB acrylic as the distributed generator material achieved a power output of up to 3.8 W in an artificial wave tank experiment, with a measured wave-to-electricity conversion efficiency of 18%.

SBM Offshore proposed an attenuator concept based on S3 DE to adapt to different sea conditions and achieve effective energy capture by adjusting the natural frequency [57]. The S3 prototype achieved 80% radial strain and 2-W peak power output in tests. Although only small prototypes such as the buoy WEC proposed by Stanford Research Institute and Hyper Drive have been tested in the laboratory or in mild sea conditions, the results show that the power output can be significantly improved through optimization, such as increasing the starting voltage [96]. However, it remains challenging to scale up the technology from watts to kilowatts and achieve large-scale power generation. In order to make DEG a viable option for wave energy applications, key issues such as sensing and control strategies, power electronics technology, cycle life of DEG, and development of new DE materials need to be addressed [5].

Current application cases for DEG remain limited, with most focused on laboratory or short-term nearshore testing. A buoy-mounted DEG sea trial at Susaki Fishing Port in Japan demonstrated that the device could operate stably and continuously for 2 weeks in a real-sea environment, demonstrating its feasibility and energy harvesting capabilities under wave-driven conditions [97]. A dynamic model of a WEC coupled to a DEG was established, and the consistency between the model predictions and experimental results was verified under flume conditions [98]. A nearshore DEG device, based on the wave pressure difference mechanism, was subjected to cyclic testing in a flume under wide-frequency and mixed sea conditions. The deformation and fatigue behavior of the material at different frequencies were monitored, but data from long-term offshore deployments remain unpublished [99]. Devices based on alternating charge drive (AC-DEG) have achieved over 10^7^ cycles (approximately 7 d of continuous operation) in laboratory conditions with virtually no degradation, demonstrating excellent fatigue durability [63]. In a field buoy energy harvesting system, a power density of approximately 99 W/m^3^ was measured under typical sea conditions, supporting the potential long-term reliability of DEGs for ocean monitoring [100].

Under real sea conditions, tests of a buoy-based DEG wave energy device demonstrated its power generation capability in open water. In another study, a coupled DEG and OWC system was systematically tested in a wave tank using a scaled-down physical model. The results showed good agreement between the numerical model and experimental observations [98]. A DEG WEC based on a pressure difference mechanism was tested in a wave tank under irregular waves and broadband excitation. The experiments quantified the device’s dynamic response and material deformation characteristics, providing data support for evaluating its adaptability and durability under different sea conditions [99]. Despite these advances, relevant reviews still point out that long-term operational data at monthly or annual scales have not yet been reported. Issues such as material electrical fatigue, seawater aging, EB, and power electronics stability remain key challenges for the large-scale application of DEG technology [101].

To provide a comprehensive overview of the current state of DEG-based WEC development, Table 8 summarizes the key experimental validations and sea trials reported in the literature, including test environments, device configurations, performance metrics, and main findings.

**Table 8.** Summary of experimental validations and sea trials for DEG-based wave energy converters

Study	Test environment	Device type	Performance	Key findings
Chiba et al. [96]	Open sea (mild conditions)	Point absorber buoy	0.25 W (avg), 1.2 W (peak), up to 11 W at higher voltage	First offshore DEG WEC demonstration
Chiba et al. [97]	Susaki Fishing Port, Japan	Buoy-mounted DEG	Stable operation for 2 weeks	Demonstrated feasibility in real-sea environment
Kornbluh et al. [94]	Laboratory/nearshore	Point absorber	>100 mJ/g cycle energy density	High energy density under dynamic conditions
Moretti et al. [98]	Wave flume	DEG coupled WEC	Model-experiment consistency verified	Validated dynamic model accuracy
Righi et al. [99]	Wave flume	Pressure difference DEG	Broadband frequency response	Characterized fatigue behavior under mixed sea conditions
Jean et al. [57]	Laboratory	S3 attenuator	80% radial strain, 2 W peak	Demonstrated adaptive natural frequency adjustment
Xu et al. [63]	Laboratory	AC-DEG	>10^7^ cycles (~7 d)	Excellent fatigue durability with no degradation
Lu et al. [100]	Field buoy system	DEG harvester	99 W/m^3^ power density	Potential for long-term ocean monitoring
VHB-based OWC [5]	Wave tank	OWC with DEG	3.8 W, 18% efficiency	Verified OWC-DEG integration performance

The DEG-based point absorber WEC uses the wave-induced interface oscillation to drive the DEG PTO system to perform periodic deformation, efficiently converting wave energy into electrical energy. DEG PTO can be used as a direct replacement for existing electromagnetic PTO systems, simplifying structural design and improving energy conversion efficiency. At the same time, the combination of DEG and elastic membrane materials helps reduce equipment wear and enhance the reliability and survivability of WECs [6]. In addition, DEG can be integrated into membrane-based WECs (mWECs), replacing the existing mechanical PTO system to simplify the WEC design and optimize the device structure.

## Dielectric Fluid Generators

### Fundamentals of DFG operation

DFG operates based on dielectric fluid-based transformer (DFT) principles. DFT is a shape-adjustable capacitor that uses a nonconductive fluid as a dielectric, which is guided to fill between 2 shape-adjustable electrodes. Figure 1C shows a cross-section of a typical axisymmetric DFT with a cylindrical chamber filled with dielectric fluid. The chamber is surrounded by a circular DE membrane with a deformable electrode attached to the upper surface of the membrane and a flat rigid circular electrode wrapped around the bottom. The upper deformable membrane is fixed by an annular support that also fixes the side walls of the fluid chamber. The membrane deflects and squeezes the bottom rigid electrode to increase the transducer capacitance.

By adjusting the flow of fluid in the cavity, the deformable electrode moves smoothly between the rigid electrodes, and the DFT is switched between a high capacitance configuration and a low capacitance configuration. In the high capacitance configuration, the contact area between the upper deformable membrane and the bottom rigid electrode is maximal. In the low capacitance configuration, the contact area is zero, and the fluid layer thickness is maximal.

In any other intermediate configuration, the thickness of the fluid layer is not uniform. When moving from a low capacitance configuration to a high capacitance configuration, the elastic membrane initially contacts the rigid electrode at a certain point, where the thickness of the fluid film is finite, while the thickness varies in other regions. As the contact area between the membrane and the electrode gradually increases, the entire electrode surface gradually contacts the membrane, making the thickness of the fluid film gradually zero in all regions.

The main advantage of DFT over DET is that it can achieve larger capacitance changes with smaller electrode and dielectric deformations. This means that for the same energy output, DFT requires lower mechanical work input and exhibits lower mechanical impedance. In addition, DFT based on dielectric fluids has the ability to self-repair dielectric breakdown, extending the service life. The reason is that the breakdown field of dielectric fluids is usually lower than that of dielectric solids, which makes it easier to establish conductive paths in dielectric fluids without the participation of dielectric solids. EB will lead to irreversible failure in dielectric solids, but not in dielectric fluids. After the transducer is deactivated, the nonconductivity of the damaged layer can be restored [102].

A DFG is an electrostatic variable capacitance transducer that is mainly composed of flexible electrodes, solid dielectric materials, and deformable dielectric fluids. It works by converting oscillating mechanical energy into direct current [103]. The flow of dielectric fluid under the action of an electric field generates charges and eventually converts these charges into electrical energy. The DFG system contains 2 electrodes, and the dielectric fluid is liquid metal, ionic liquid, etc. Figure 1C shows a schematic diagram of the DFG power generation cycle and gives the theoretical equipotential cycle of the DFG. The capacitance changes from $C_{l}$ in the flat configuration to $C_{h}$ in the maximum bending configuration. $C_{c}$ and $W_{c}$ represent the “contact” configuration, where the membrane is in contact with the plate at only one point.

The power generation cycle of DFG has 4 key stages, including 2 equipotential processes and 2 isochoric processes. The voltage *V* is the potential difference between the electrodes, and Ω is the volume of the fluid in the variable capacitor cavity. The theoretical equipotential cycle of DFG contains 2 equipotentials, as shown and represented by red solid lines in Fig. 1C. The 4 stages of the power generation cycle are as follows:

1. From 1 to 2 is the capacitance increase stage. Under constant zero voltage, the negative fluid pressure pushes the elastic membrane to bend and squeeze the rigid plate electrode, resulting in an increase in the DFG capacitance. Mechanical work is transferred from the fluid to the membrane and stored as elastic energy.
2. From 2 to 3 is the charging stage. While keeping the volume of the fluid in the cavity unchanged, opposite charges are transferred to the 2 electrodes until the target voltage is reached. An external power supply provides charges to the electrodes, and part of the electrical energy is transferred to the DFG through the external power supply and stored in the form of electrostatic energy.
3. From 3 to 4 is the energy generation stage. As the fluid pressure increases, the elastic membrane returns to its undeformed state under the action of the fluid pressure while the voltage remains unchanged, resulting in an increase in the distance between the electrodes and a decrease in the DFG capacitance. Part of the charge on the electrodes is released, the elastic energy is recovered, and the mechanical work and charging energy are transferred out of the system.
4. From 4 to 1 is the discharge phase. While maintaining the volume of the fluid in the chamber constant, the charge is gradually removed from the electrodes until the voltage drops to zero. The remaining electrostatic energy is discharged and removed from the system [103].

### Key materials and system architecture

DFT is a core element in the DFG system architecture. It is a variable capacitance electric transducer composed of flexible electrodes, solid dielectrics, and variable volume dielectric fluids. It changes capacitance through mechanical input to achieve bidirectional conversion of mechanical energy and electrical energy. The designed DFT prototype adopts the cCFr architecture, including rigid metal electrodes, flexible electrolyte membranes coated with conductive carbon grease, and dielectric fluids (such as silicone oil). The capacitance value can be significantly adjusted by controlling the inflow and outflow of dielectric fluids. The laboratory test results show that the prototype has a maximum output power of 0.575 mW in each energy conversion cycle, can convert up to 4.6 mJ of energy, and has a demonstrated conversion efficiency of 30%, showing good repeatability and stability in multiple cycle tests. The energy conversion density per unit mass of DFG in dielectric fluids and solid media is 63.8 and 179.0 mJ/g, respectively, showing great potential for energy harvesting [103].

DFG performance is calculated as the product of the relative permittivity of the fluid and its dielectric strength, which represents the maximum displacement field that the material can withstand before EB occurs. The maximum energy density of the DFG is governed by a second-order power law. When the dielectric properties of the fluid are weak, the energy density of the DFG decreases, although the high permittivity of the elastic film allows lower operating voltages and maintains good performance. Therefore, adding an additional solid dielectric layer is a reasonable compromise when voltage is strictly required. Since real dielectrics always have a finite resistivity, the DFG suffers from charge losses from one electrode to the other during operation. To reduce leakage losses, it is necessary that the cycling frequency is much higher than the typical discharge time, and for each *k*th dielectric material in the stack, $\tau_{k}=\rho_{k}\epsilon_{k}$, where $\rho_{k}$ is its resistivity. If the characteristic discharge time between 2 adjacent dielectric layers differs greatly, static surface charge accumulates at the interface, which is calculated as:

$$\sigma_{c}=\frac{V}{R_{t}h_{t}}\left(\tau_{s}-\tau_{f}\right)$$ (9)

where $\sigma_{c}$ is the charge density on the contact interface under steady-state conditions; $R_{t}$ and $h_{t}$ are the total resistance and height of the dielectric stack, respectively; *V* is the total voltage across the stack; and $\tau_{s}$ and $\tau_{f}$ are the discharge constants of the solid and fluid dielectric layers, respectively. This surface charge should be avoided as it can lead to transient leakage phenomena that are difficult to predict.

The electrical properties of the materials used to make the DFG flexible and fluid layers are summarized as follows [103–105]. Solids for the deformable membrane include NR, synthetic rubber, silicone rubber, and acrylic rubber. Rigid mica and glass are also options for implementing DFG architectures that require rigid solid dielectrics, presented in Fig. 1C. Fluids considered include mineral, silicone oil, and insulating esters, which are widely used in the transformer insulation industry. They are designed to have high dielectric strength, but their dielectric constants are relatively limited.

Fluid materials include silicone oil, mineral oil, and ester. Silicone oil has a relative permittivity of 2.7, a dielectric strength of 30 to 45 MV/m, a breakdown displacement field of 81 to 121 MV/m, an electrical resistivity of 10^13^ Ω·m, and a discharge constant of 23.9 s. Mineral oil has a relative permittivity of 2.2, a dielectric strength of 39 MV/m, a breakdown field of 86 MV/m, an electrical resistivity of 10^11^ Ω·m, and a discharge constant of 1.9 s. Ester has a relative permittivity of 3.2, a dielectric strength of 45 MV/m, a breakdown field of 144 MV/m, an electrical resistivity of 3 × 10^10^ Ω·m, and a discharge constant of 0.8 s.

Elastomer materials include NR, synthetic rubber, silicone rubber, and acrylic rubber. NR has a relative permittivity of 2.7, a dielectric strength of 100 to 300 MV/m, a breakdown displacement field of 270 to 810 MV/m, an electrical resistivity of 10^12^ Ω·m, and a discharge constant of 23.9 s. Synthetic rubber also has a relative permittivity of 2.7, a dielectric strength of 100 to 300 MV/m, a breakdown field of 270 to 810 MV/m, an electrical resistivity of 10^12^ Ω·m, and a discharge constant of 47.8 s. Silicone rubber has a relative permittivity range of 1.5 to 2.8, a dielectric strength of 70 to 150 MV/m, a breakdown field of 105 to 420 MV/m, an electrical resistivity of 10^12^ Ω·m, and a discharge constant of 13.3 s. Acrylic rubber has a relative permittivity of 2.9 to 4.1, a dielectric strength of 60 to 180 MV/m, a breakdown field of 168 to 738 MV/m, an electrical resistivity of 10^11^ Ω·m, and a discharge constant of 2.6 s.

Solid layer materials include mica and glass (Pyrex). Mica has a relative permittivity of 3.0 to 6.0, a dielectric strength of 118 MV/m, a breakdown displacement field of 354 to 708 MV/m, an electrical resistivity of 10^13^ Ω·m, and a discharge constant of 265.5 s. Glass (Pyrex) shows a relative permittivity of 3.7 to 10.0, a dielectric strength of 9.8 to 13.8 MV/m, a breakdown field of 36 to 138 MV/m, an electrical resistivity of 10^12^ Ω·m, and a discharge constant of 32.7 s [103].

A nonuniformity factor of 1.5 was applied when converting the dielectric strength values from the spherical tests to those from the flat tests [105]. Preliminary chemical compatibility between commercially available elastomers and the fluids was evaluated by observing the effects of dropping a fluid on the elastomer surface for 24 h. Based on the test results in Table 9, silicone oil is inferred to be compatible with all tested elastomers except silicone rubber. Acrylic elastomers adsorb mineral oil and esters with significant effects within 24 h. Although esters have good chemical compatibility with elastomers, their low discharge constants limit their energy generation efficiency in most practical applications. Simultaneous testing showed significant energy leakage losses in the frequency range from 0.1 to 1 Hz. Among the fluids studied, silicone oils were determined to be the ideal choice for achieving the best balance between electrical performance and compatibility with common elastomers. The development and adoption of dielectric fluids designed specifically for DFG technology is a promising development.

**Table 9.** Summary of the preliminary test of chemical/physical compatibility between dielectric fluids and dielectric elastomers

Elastomer	Fluid		
	Silicone oil	Mineral oil	Ester
	Xiameter 200 50st	IP Ditrans CK	MIDEL 7131
Synthetic theraband	Effects: None Usable: Yes	Effects: Chemical or physical alterations Usable: No	Effects: Chemical or physical alterations Usable: No
Natural rubber Oppoband	Effects: None Usable: Yes	Effects: Chemical or physical alterations Usable: No	Effects: Chemical or physical alterations Usable: No
Silicone Elastosil Wacker	Effects: Chemical or physical alterations Usable: No	Effects: Chemical or physical alterations Usable: No	Effects: None Usable: Yes
Acrylic VHB 3M	Effects: None Usable: Yes	Effects: Minor/slow interaction Usable: Uncertain	Effects: Minor/slow interaction Usable: Uncertain

### Performance characteristics and efficiency

The long-term stability and durability of DFG are mainly affected by fatigue strength, ductility, and dielectric fluid properties. Compared with DEG, the dielectric fluid of DFG shows less deformation under the action of electric field, has lower fatigue performance, and exhibits good ductility and adaptability. Its manufacturing and maintenance costs affect its market competitiveness to a certain extent [67,103]. DFG relies on excellent tensile properties to withstand large deformations, enhancing its reliability under different electric field conditions. The pre-stretching value affects the operation of the prototype and improves its working stability [103]. Because of the use of dielectric fluid materials known to be resistant to seawater corrosion, DFG has potentially better corrosion resistance in marine environments than DEG. For example, NR, a material often used for membranes in DFG prototypes, has been used in harsh environments such as the ocean for more than a century and has shown good reliability, suggesting that DFG could be well adapted to the marine environment even under low strain conditions [8]. Therefore, to enhance durability, the use of tough and corrosion-resistant films, such as commercial NR elastomers [5], becomes an effective solution.

Although the manufacturing cost of DFG is higher than that of DEG, especially when high-performance dielectric fluid materials are used, its high deformation capacitor made of elastic dielectric and adapted electrodes provides better energy density and excellent conversion efficiency, so it still has a competitive advantage in characteristic application scenarios [103]. In the marine environment, DFG must have good seawater corrosion resistance and anti-interference ability to ensure the normal operation of the equipment, and its working efficiency directly affects the effect of energy conversion. In order to extend the service life of DFG and ensure the long-term reliable operation of the equipment, the equipment must always be kept within a reasonable working load range to avoid material damage due to extreme stress [8,103]. Since the flow of dielectric fluid is relatively easy to achieve, DFG maintains higher working efficiency during energy conversion, which mainly depends on the maximum energy density and overall energy conversion efficiency in each working cycle. A key strategy to improve the overall efficiency of DFG is to reduce the duty cycle so that more energy can be converted per unit time. In the experiment, the capacitance change of the device is related to the theoretical expected value, but there is a certain deviation due to the incomplete discharge of the fluid between the electrodes. The loss of conversion energy increases with the electric field and is proportional to the duration of the generation phase. Charge leakage is one of the main factors causing energy losses, while fluid viscosity losses caused by imperfect system fluid dynamics design also limit efficiency [103].

### Case studies of DFG applications

The research team built a complete DFG prototype experimental platform to verify its performance and optimize related parameters [103]. The experimental platform consists of a DFG prototype, a hydraulic subsystem, a high-voltage electronic subsystem, and a real-time controller. The DFG prototype is connected to the liquid flow regulation system through a hydraulic piston driven by a linear motor to control the volume of the discharged fluid. The high-voltage driver is responsible for electrical actuation, and 3 pressure sensors are used to monitor the pressure changes in the fluid chamber in real time. The entire system is controlled by a real-time target drone. The DFG prototype used adopts a cCFr topology structure, and the core part is a closed fluid chamber. The bottom of the fluid chamber is made of a rigid brass plate, the side wall is an annular fluid distributor made of acrylonitrile butadiene styrene (ABS) material, and the top is closed with an elastic dielectric diaphragm and fixed by an ABS annular bracket. All ABS parts are made by 3D printing, dried after acetone vapor treatment to ensure their waterproof performance. Figure 1C shows 4 dielectric fluid sensor (DFT) architectures. In cCFr and cCFCc [Fig. 1C (i) and (ii)], the flexible electrodes are located outside the sensor, mounted on a DE support layer. In cFRr and cFRFc [Fig. 1C (iii) and (iv)], the electrodes are made entirely of conducting polymers and isolated by a rigid dielectric layer [103].

Xiameter PMX-200 50CS was selected as the dielectric fluid. This silicone oil has been shown to significantly improve electrical performance when used in conjunction with DET [106]. The fluid chamber ring distributor has 3 key roles: First, it is able to evenly distribute the fluid from the inlet and outlet pipes to the center of the chamber, ensuring stable fluid flow. Second, it maintains a fixed spacing between the membrane support and the brass electrodes, ensuring uniformity of the electric field and stable capacitance changes. Third, it provides 3 side openings spaced 60° apart to connect pressure sensors for real-time monitoring. The brass electrodes are firmly bonded to the distributor with acrylic sealants, the top is closed by an elastic membrane, and the external flange and screws firmly fix the device to ensure the oil tightness of the system. The inner diameter of the ring and brass plate is 150 mm, and the distance between the electrodes in the flat position is 4.5 mm.

The elastic membrane was made by gluing together 3 layers of commercial double-sided pressure-sensitive acrylic tape VHB 4905, each with an original thickness of 0.5 mm, which were pre-stretched to 3 times and fixed on a ring bracket to provide the necessary prestress. After multiple rounds of test optimization, the pre-stretch value was determined to ensure the smooth operation of the device during the generation phase, avoid the delay of membrane separation caused by too low pre-stretch value, and ensure that the DE can be smoothly separated from the brass electrode when the fluid impacts the transducer. The maximum stretch applied was 1.12 times, resulting in a total maximum stretch of 3.36 times, which is much lower than the fracture limit of VHB material [107]. Therefore, this pre-stretch value is considered to be the optimal choice for the normal operation of the device. The use of harder elastic dielectric materials can appropriately reduce the degree of pre-stretching. The use of deformable electrodes and the coating of carbon conductive grease on the upper surface of the elastic film ensure good conductivity (MG-Chemicals 846) [108].

Based on the dielectric constants of silicone oil ($\epsilon_{f}$ = 2.7) and acrylic elastomer ($\epsilon_{s}$ = 4.15), the minimum capacitance ($C_{l}$) of this DFG prototype is calculated to be 92.0 pF and the maximum capacitance ($C_{h}$) is 2.34 nF. It is assumed that the capacitance reaches a minimum when the membrane surface is completely flat. The capacitance can be maximized by introducing a deformed morphology of the membrane and optimizing it under the minimum dielectric oil volume condition. In the model, the part of the membrane in contact with the brass electrode remains flat, the part of the membrane not in contact with the electrode is annular, and the separated membrane part has a constant radius of curvature in the meridian cross section and is connected to the flat part by a horizontal tangent. Given the geometry and considering the maximum fluid volume displacement (Ωf) of 67.1 cm^3^, the shape of the membrane can be defined, and the capacitance value can be approximately calculated. The results show that the contribution of the membrane part in contact with the brass electrode to the total capacitance reaches 86%, which is significantly higher than the contribution of the uncontacted part and has a stronger correlation with the remaining annular area. Therefore, optimizing the contact area between the diaphragm and the electrode becomes a key factor in improving the performance of DFG. When the dielectric strength of the fluid is 45 MV m^−1^, keeping the above parameters and the expected capacity, the theoretical maximum electrical output energy of DFG is estimated to be 17.98 mJ/cycle [103].

In order to achieve precise control of the fluid, the DFG prototype was connected to a hydraulic system in the experiment. The system is equipped with an E200-907 oil injector produced by Expert-tool, and the drive device uses a brushless linear motor of LinMot’s P01-37×120F/200×280-HP. A closed-loop feedback controller is installed in the motor driver to accurately adjust the piston position and ensure the stability of fluid input and output to ensure consistency of experimental conditions. In terms of fluid management, the inlet and outlet pipes of the hydraulic system are equipped with a pressure relief valve to exhaust the air in the hydraulic system. The connection between the prototype, the ejector, and the valve is completed through a semiflexible silicone tube with an inner diameter of 7 mm. This material provides sufficient flexibility to adapt to the experimental environment and effectively reduces the energy loss during fluid transportation. The system is integrated with 3 high-precision pressure sensors (P1, P2, and P3), which are connected to the DFG side port through a thin silicone tube to form a small air buffer, thereby reducing the impact of fluid transient pressure fluctuations on measurement accuracy. During the experiment, the sensor detects the oil pressure difference in real time [103] and compares it with the oil pressure difference set at the beginning of each test to monitor the performance of DFG under different working conditions in real time.

Important breakthroughs have been made in the study of DFG’s electromechanical conversion mechanism. By adopting an innovative dielectric fluid–electrode coupling structure [109], DFG successfully achieved efficient electromechanical energy conversion. Under external mechanical excitation, the displacement of the dielectric fluid causes the system capacitance to change, thereby generating electrical energy output [103]. When EB occurs in the dielectric fluid, the DFG system, which has a “self-healing” property, can automatically recover without causing permanent failure [109]. The maximum operating voltage of DFG is determined by the breakdown displacement of the dielectric material, among which the combination of mineral oil and acrylic elastomer shows the best performance. Compared with traditional DEGs, the elastic energy storage fluctuation of DFG is reduced by about 70%, which gives it a significant advantage in applications such as wave energy collection [95].

The experimental performance of DFG shows excellent potential for practical applications. The pumped fluid DFG prototype developed by the research team has achieved innovative performance indicators: Single-cycle energy conversion is increased to 4.6 mJ, peak power reaches 0.575 mW, and energy conversion efficiency is as high as 30% [103]. Experimental studies have shown that the performance of DFG is constrained by 3 core factors: (a) The viscosity of the dielectric fluid directly affects the response speed. (b) The flexibility of the electrode material affects the energy conversion efficiency. (c) The uniformity of the dielectric layer thickness determines the stability of the system. This study quantitatively analyzed the capacitance variation range of DFG for the first time and confirmed that its maximum capacitance ratio can reach 10:1, providing an important basis for optimizing energy collection efficiency. DFG maintains stable output in the operating frequency range of 20 to 50 Hz, which makes it particularly suitable for dynamic application scenarios such as human motion energy recovery [109].

Compared to solid thin-film DEGs, DFGs have been proposed for wave energy capture due to their liquid dielectric self-healing and large deformation properties. Essentially, DFGs are variable capacitors based on a moving liquid medium, capable of maintaining high energy conversion efficiency at low frequencies [110]. Early research has constructed an experimental platform comprising hydraulic actuation and high-voltage electronics, demonstrating single-cycle energy outputs of tens of millijoules and energy conversion efficiencies approaching 30% under various hydraulic excitations, demonstrating the feasibility of this technology under laboratory conditions [103]. Subsequent work has proposed a hydraulically amplified self-healing electric actuator (HASEL), which uses a fluid–electrode coupling mechanism similar to that of DFGs. Experimental results have demonstrated self-healing and long-term reliability under high-voltage cyclic excitation, providing insights into improving device durability [109]. Experiments under controlled wave tank conditions further demonstrated a wave energy harvesting prototype based on dielectric fluids, achieving high power density under resonant conditions and demonstrating the potential of DFG for ocean energy capture [95]. Recent studies have shown that liquid dielectric devices using hydraulic amplification and self-healing mechanisms (such as HASEL structure) have better impact resistance and durability in complex environments [111]. A related review systematically summarizes the modeling, optimization, and experimental progress of DFG/DEG systems and points out that the key to the future lies in transitioning from laboratory prototypes to pilot-scale and long-term sea trials [5].

The modeling and optimization of DFG has achieved a major breakthrough. The research team has built a new multi-scale modeling method to effectively predict the complex electromechanical coupling characteristics of DFG. For the Peano HASEL structure, the model successfully captures its nonlinear force–displacement characteristics, with a prediction error of less than 5%. The researchers introduced the Gent hyperelastic model and systematically characterized the mechanical behavior of dielectric films under large deformation conditions for the first time [112]. Numerical simulations reveal a key phenomenon: At a specific voltage, the pressure–volume curve of DFG exhibits a nonmonotonic characteristic, suggesting that the system may have a bistable characteristic. Based on these research results, the study proposed the optimization design criteria for DFG:

1. Use ester fluids with high dielectric constants.
2. Precisely control the pre-stretching ratio of the dielectric layer.
3. Optimize the electrode layout to maximize the capacitance change.

These research results provide solid support for DFG’s application in the field of renewable energy, especially in wave energy collection and micro-energy systems, showing broad application prospects [95,103].

## Comparative Analysis of DEGs and DFGs

Energy harvesting in marine environments presents unique challenges that demand specialized technologies. This section provides a detailed comparative analysis of 2 promising approaches: DEGs and DFGs. By examining their respective strengths, weaknesses, and performance metrics, we can better understand their suitability for offshore energy harvesting applications.

Recent advances in material selection, energy recycling pathways, and power electronics matching have significantly improved the performance of both DEG and DFG technologies. Table 10 provides a comprehensive summary of experimentally reported performance metrics for both technologies, compiled from recent literature. As shown in the table, DEGs have demonstrated remarkable energy densities ranging from 111 mJ/g for AC-driven systems to as high as 780 mJ/g with optimized triangular looping strategies. The conversion efficiencies have also seen substantial improvements, with AC-DEGs achieving single-cycle efficiencies of 51.8% and integrated systems reaching peak efficiencies approaching 60% under optimized conditions.

**Table 10.** Quantitative performance comparison of dielectric elastomer and dielectric fluid generators

Metric	Category	DEG	DFG
Energy density			
	Experimental	1.7 J/g (acrylic) [185], 780 mJ/g (triangular) [43], 560 mJ/g (equibiaxial) [43], 130 mJ/g (pre-stretch) [43], 111 mJ/g (AC-DEG) [63], range: 0.10–0.78 J/g [5]	0.064–0.179 J/g [103], 35.2 μW/g
	Theoretical	1.7–3.5 J/g [186,187]	Max. 17.98 mJ/cycle [103]
	Charge density	26 mC/m^2^ [188]	–
Conversion efficiency			
	Experimental	51.8% (AC-DEG) [63], ~60% (peak) [189], >40% (integrated) [189], 7.2% (lower) [38]	~17.8% [190]
	Theoretical	80–90% [43]	~30% [103]
Durability			
	Experimental	>10^7^ cycles (AC-DEG) [63], 1.73 × 10^7^ (silicone) [29], >10^6^ (wave) [98]	10^4^−10^5^ cycles, short-term only [103]
Power output	Experimental	–	1.83 mW (max) [190]
Cost	Material	$6–100/kg [5,191]	Higher (complex system) [103]
Scalability	Principle	Excellent (modular/roll-to-roll) [31]	Good (modular, no data) [103]

In terms of fatigue life, DEGs have shown exceptional durability, with systematic studies demonstrating stable operation exceeding 10^7^ cycles under controlled laboratory conditions (see Table 10). This represents a significant advancement for long-term reliability, particularly in ocean wave energy applications where devices must withstand continuous cyclic loading. In contrast, DFG technology, while showing promise in terms of self-healing capabilities and corrosion resistance, currently lacks comparable long-term fatigue data, with existing prototypes limited to shorter operational periods.

The performance comparison in Table 10 reveals critical trade-offs between the 2 technologies. While DEGs excel in energy density and demonstrated fatigue life, DFGs offer advantages in terms of lower mechanical impedance and self-healing dielectric properties. These differences have important implications for technology selection in specific offshore deployment scenarios, as discussed in the following subsections.

### Strengths and weaknesses of both technologies

#### Dielectric elastomer generators

DEGs possess several notable strengths. They exhibit excellent ductility, allowing them to accommodate large deformations and making them highly adaptable to varying mechanical inputs [113]. The manufacturing costs of DEGs are relatively low, as their primary materials consist of commonly available rubber or elastomeric polymers [8]. DEGs also offer high scalability, capable of being manufactured in various sizes and configurations to suit different applications [49]. The compatibility of DEGs with low-cost materials and processes, along with lightweight sensors and actuators, further enhances their appeal for certain applications. Additionally, their high deformation capacity makes them particularly valuable in applications where significant shape changes are required.

However, DEGs also present significant weaknesses. Their operating principle involves cyclic deformation of the elastomer, leading to high fatigue susceptibility and limited lifetime [8]. DEGs demonstrate lower stability compared to alternative technologies, requiring additional control mechanisms to ensure consistent performance [49]. The mechanical properties of elastomers used in DEGs are significantly affected by environmental conditions, particularly temperature and humidity. Significant hysteresis due to high energy dissipation necessitates high electric fields for operation. Additionally, the large strains involved cause electrode fatigue, requiring regular replacement of elastomeric components. These limitations can be particularly problematic in applications requiring long-term reliability without frequent maintenance.

#### Dielectric fluid generators

DFGs offer distinctive advantages compared to DEGs. They demonstrate superior fatigue resistance due to the flow-based charge movement within the dielectric fluid, enhancing long-term operational reliability [114]. The ability to select dielectric fluids with excellent seawater corrosion properties makes DFGs particularly suitable for marine applications [115,116]. DFGs exhibit greater stability in environments with external interference, making them appropriate for applications requiring high operational stability. Dielectric fluids possess self-healing capabilities, allowing them to recover quickly after breakdown and restore their insulating function, although gradual degradation may occur over time. DFGs require less deformation for electrodes and dielectric to obtain large capacitance changes, resulting in lower mechanical work input for equivalent energy output.

These anti-breakdown characteristics effectively prevent breakdown phenomena, thereby enhancing equipment stability and reliability, which is a crucial advantage in long-term deployments. DFGs’ energy conversion efficiency is exceptional, effectively transforming mechanical vibrations or pressure variations into electrical energy, providing an efficient energy conversion mechanism with extensive application potential. The deformable structure of DFGs incorporates solid materials with flexible films, such as elastomers, granting them considerable structural adaptability and enhancing their flexibility under varying environmental and application requirements. As self-powered generation systems, DFGs operate without external power sources, making them ideal for deployment in locations where traditional power infrastructure is unavailable or impractical.

Despite these advantages, DFGs have several limitations. They are generally more expensive to manufacture, particularly when high-performance dielectric fluids are utilized [114,116]. The performance of DFGs heavily depends on selecting appropriate dielectric fluids for specific application scenarios, increasing system design complexity [115]. The frequency stability of DFGs is strongly affected by temperature and environmental changes. DFGs involve sophisticated material selection and manufacturing processes, leading to challenges in maintenance and potential increases in operational costs. Energy losses due to fluid viscosity may be significant, potentially limiting the bandwidth of DFGs in certain drive and power generation applications. These disadvantages must be carefully weighed against their benefits when considering specific deployment scenarios.

### Performance metrics: Fatigue resistance, ductility, corrosion resistance, cost, and efficiency

While the previous sections outlined the technologies’ principles, a quantitative comparison is essential for a practical assessment. Table 10 provides a consolidated summary of key performance metrics for both technologies, compiled from recent literature and distinguishing between experimental and theoretical data to provide a stronger comparative foundation.

The quantitative data presented in Table 10 allow for a direct, evidence-based comparison of the 2 technologies, revealing critical trade-offs and highlighting their current technological status. A primary insight is the significant gap between theoretical potential and experimental reality, particularly for DEGs. While theoretical models predict extraordinary energy densities for materials like acrylics and rubber (up to 3.5 J/g), the highest reported experimental value is 0.78 J/g. Similarly, the experimental efficiency (up to 51.8%) is impressive but still far from the theoretical 80% to 90%. This suggests that current experimental setups and material implementations are only able to unlock a fraction of the technology’s intrinsic potential. In contrast, DFG technology shows a closer, albeit more modest, alignment between its theoretical and experimental performance, indicating that its challenges may lie more in scaling than in fundamental material limitations.

The data on durability in Table 10 underscore a critical barrier for commercialization. The best-reported experimental fatigue life for DEGs, over 10^7^ cycles [63], represents a significant laboratory achievement. However, as will be discussed in detail in System-level integration and engineering challenges, this is still 1 to 2 orders of magnitude below the requirements for long-term offshore structures, highlighting a fundamental material science challenge. The explicit lack of long-term fatigue data for DFGs is itself a key finding from the literature, pointing to its status as a more nascent technology and a critical area for future investigation.

### Analysis of theoretical models under marine conditions

#### Electromechanical coupling model

Electromechanical coupling models describe the interaction between electric fields and large-deformation elastomers based on nonlinear elasticity theory and Maxwell stresses. However, existing studies often assume that the material is ideally dry and maintained at a constant temperature, with uniform properties [101]. In reality, electrode preparation, temperature, and humidity can significantly affect the breakdown strength. For example, studies on the EB behavior of silicone rubber films using different electrode preparation methods show that the breakdown field strength decreases significantly under high humidity conditions. With a carbon black electrode, the breakdown field strength at 90% relative humidity (RH) is approximately 3% lower than that at 10% RH, while with a metal electrode (at 80 °C), the decrease reaches 13.5% [117]. The breakdown voltage decreases significantly at higher temperatures, with some silicone elastomer samples experiencing a drop of up to 30% between 20 and 80 °C [118]. Furthermore, the electrical and mechanical properties of DEs are highly sensitive to environmental conditions. Increased temperature alters the mechanical response of PDMS, while high humidity significantly reduces the breakdown field strength and dielectric properties, limiting its electromechanical coupling performance and accelerating performance degradation [119].

**Fig. 4.**
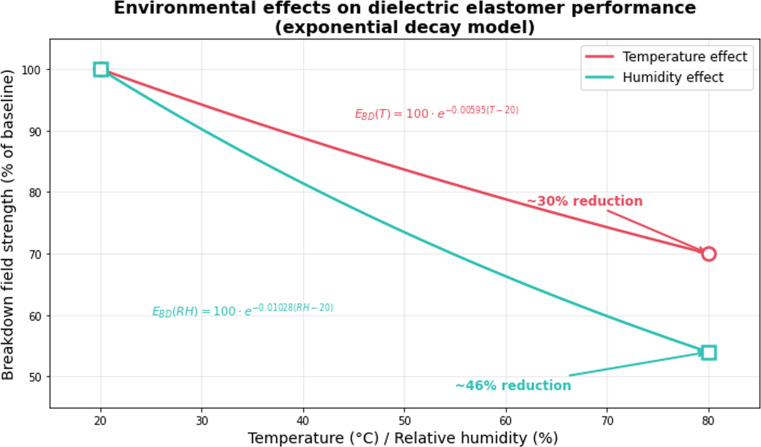
Environmental effects on dielectric elastomer performance showing exponential decay models for temperature and humidity impacts on breakdown field strength.

The electrical properties of VHB elastomers are also affected by humidity. When the humidity increases from 20% RH to 80% RH, the breakdown field strength decreases significantly, and high humidity weakens its insulation properties [120]. Increased humidity increases the hysteresis effect and conductivity, resulting in a decrease in equivalent capacitance and electrodeformation efficiency [121]. The breakdown strength of silicone rubber decreases after electromechanical cycling, especially during the first stretching cycle, where the average breakdown strength decreases by approximately 30%. Subsequent cycles have little effect on further degradation of the breakdown strength [119]. High humidity and moisture absorption in marine environments can significantly degrade the insulating properties of silicone-based DEs and accelerate electrochemical or structural degradation [118]. The environmental degradation of DE performance follows an exponential decay model, as shown in Fig. 4, where temperature effects result in approximately 30% reduction in breakdown field strength and humidity effects cause up to 46% reduction under extreme conditions. Furthermore, wave loads in marine environments exhibit irregular and multi-frequency characteristics, while theoretical models and scaled-down experiments are often based on the assumption of regular waves, resulting in discrepancies between actual output power and theoretical predictions [101].

#### Fatigue lifetime model

Most existing fatigue and breakdown models are based on idealized or constant-load conditions. However, for elastomeric materials like PDMS, their actual mechanical response is significantly affected by multiple factors, including time, temperature, strain rate, and loading history. This means that ignoring complex environmental and random loading effects can lead to optimistic life predictions [122]. In ocean energy environments, loading frequency and amplitude are significantly affected by irregularities in the wave spectrum. Temperature and humidity fluctuations also alter the material’s crack growth behavior, leading existing models to often overestimate life predictions [119]. In studies of silicone rubber and similar rubber materials, crack growth rates increase by approximately 2 to 5 times when the temperature increases from 25 to 75 °C. Furthermore, plasticization due to humidity or water absorption can sometimes reduce tear energy by 20% to 50%, accelerating crack growth [123].

In addition to loading conditions, environmental factors themselves can also have a significant impact on life. Electrode corrosion or surface defects can promote early crack initiation, and environmentally induced effects are often ignored in traditional fatigue models [117]. Under the same mechanical driving force, the crack growth rate in oxygen- and water-containing environments is more than 10 times higher than in dry air [124]. At the same time, water absorption significantly reduces the resistivity and breakdown strength of the material and increases the dielectric constant [125]. Under dry conditions, the bulk conductivity of silicone rubber can be as low as approximately 10^−14^ S/m. However, after water absorption, the conductivity increases significantly, and the DC breakdown strength decreases by approximately 10% to 20% [126].

#### Dielectric breakdown model

Dielectric breakdown models define the safe operating range of a device using the breakdown electric field strength, but this parameter is often idealized at dry room temperature [118]. In practical applications, ambient humidity significantly affects the breakdown performance of DEs. Experimental results show that increasing the RH from 20% to 80% reduces the breakdown field strength by approximately 46%. The primary mechanism for this is that high humidity facilitates the formation of conductive pathways within the material and at the electrode interface [127]. Furthermore, the breakdown behavior of DEs depends not only on the bulk properties of the material but also on the combined strain, edge defects, and electrode geometry and surface morphology. These factors can lead to localized electric field concentrations that trigger breakdown at the surface or electrode edge, rather than the uniform bulk breakdown mode assumed in the model [119].

### Suitability for offshore energy harvesting

When selecting between DEGs and DFGs for offshore energy harvesting, it is essential to consider the unique challenges of the marine environment, including corrosive seawater, variable mechanical inputs, and the need for reliable long-term operation with minimal maintenance [114,115]. The specific application scenario plays a crucial role in this decision-making process.

DEGs are more suitable for applications where significant deformation capabilities are required but long-term stability demands are relatively lower [8]. In these scenarios, the flexibility and adaptability to deformation are paramount considerations while compromising somewhat on long-term structural stability. This makes them potentially viable for short-term deployments or prototype testing in marine environments, especially when budget constraints are a primary concern [49].

In contrast, DFGs demonstrate superior performance in applications that demand resistance to seawater corrosion and require continuous, reliable operation over extended periods [114,116]. Their exceptional performance in harsh environmental conditions makes them particularly valuable for offshore deployments where maintenance access is limited and system reliability is critical. Some systems may even be adapted for motion monitoring and wireless control applications [128], enhancing their versatility in marine settings. The selection of DFGs for energy harvesting applications is justified by several compelling factors. They offer exceptionally high-performance energy conversion capabilities, as dielectric fluids can move in complex patterns when subjected to electric fields, resulting in enhanced mechanical energy capture. Variable capacitors within these systems can efficiently convert mechanical energy into electrical energy bidirectionally.

DFGs exhibit remarkable flexibility, moving freely within liquid environments and adapting to diverse shapes and conditions without the constraints imposed by rigid mechanical connections. Their application versatility spans numerous domains, from harnessing ocean tidal energy to capturing fluid movement in liquid pipelines. Unlike solid dielectric materials, EB in liquids does not result in permanent failure but merely causes a brief functional interruption, significantly enhancing their reliability in critical applications.

The environmental resilience of DFGs makes them well-suited for prolonged deployment in offshore conditions. Their excellent seawater corrosion resistance and strong anti-interference capabilities allow them to withstand the harsh marine environment, including fluctuating temperatures and mechanical stresses from wave action. While certain elastomer materials used in DEGs can be adapted for seawater exposure, their performance is typically optimal only under low-strain conditions, limiting their durability in highly dynamic marine settings.

The extended operational lifespan of DFGs presents a significant advantage, particularly in offshore applications where maintenance access is costly and infrequent. Their lower fatigue characteristics contribute to longer service life, reducing the need for frequent replacements. In contrast, DEGs, which rely on elastomer materials prone to fatigue, require more frequent maintenance, presenting logistical challenges in marine deployments.

Energy conversion efficiency is another critical factor, and DFGs generally outperform DEGs in this regard. Their superior energy harvesting capabilities allow for more effective capture of wave and tidal energy, maximizing output in offshore environments. Additionally, their lower mechanical impedance enables a more responsive interaction with the varied mechanical inputs characteristic of ocean waves, further enhancing efficiency.

The ability to adapt to wave dynamics is crucial for offshore energy harvesting systems. The 3D nature of ocean waves requires technologies that can efficiently respond to complex motion. While DEGs offer high ductility and can accommodate large deformations, DFGs provide better adaptability to complex shapes and fluid flow environments without the need for intricate mechanical linkages. This inherent flexibility simplifies system design, making DFGs an attractive option for marine applications.

Despite these advantages, practical implementation must also consider the higher manufacturing costs and maintenance complexities associated with DFGs. Additionally, DFGs often feature simpler designs without complex mechanical components, potentially reducing maintenance requirements and associated costs while maintaining high energy density and conversion efficiency. DFGs demonstrate superior adaptability to liquid environments such as oceans and rivers, where traditional DE actuators might face significant limitations. They can conform to complex 3D shapes and flow environments, providing distinct advantages in specialized applications like marine ecological monitoring systems that must adapt to oceanic topography.

In conclusion, both DEGs and DFGs offer viable solutions for offshore energy harvesting, but DFGs provide compelling benefits in terms of longevity, efficiency, and resilience to marine conditions [114–116]. For long-term offshore energy harvesting applications, the superior durability, efficiency, and corrosion resistance of DFGs likely justify their higher initial investment. Management considerations for implementation may also influence technology selection [129], particularly when integrating these systems into existing energy infrastructure. Energy density functional approaches may provide additional insights into optimizing these systems, potentially leading to enhanced performance in specialized applications. Future research should focus on addressing remaining challenges, particularly reducing manufacturing costs and simplifying maintenance processes for DFGs, while also exploring hybrid systems that might leverage the advantages of both technologies for specific deployment scenarios. These advancements will further enhance the potential for large-scale deployment of these promising technologies in offshore energy systems.

## Recent Advances and Future Directions

### The economic challenge: An LCOE perspective

These interconnected performance and cost trade-offs, particularly the durability deficit, directly impact the economic viability of both technologies. This is best understood through the lens of the LCOE. While a precise LCOE for nascent DG technologies is not yet available, a comparative analysis against established benchmarks is insightful. As systematically reviewed by Guo et al. [130], the projected LCOE for conventional WECs is high, with estimates ranging from $120/MWh to $470/MWh. This is significantly higher than mature marine renewables like offshore wind.

It is important to note that these LCOE estimates typically encompass the total system cost, including not only the WEC device itself but also the balance-of-plant (BoP) components such as mooring systems, power cables, grid connection infrastructure, and installation costs. For conventional WECs, the BoP components (including mooring, electrical systems, and installation) can account for 40% to 60% of the total capital expenditure (CAPEX) [88,130].

The primary reason for this cost disparity lies in the capital and operational expenditures (CAPEX/OPEX). For instance, a recent techno-economic analysis by Gao et al. [131] shows that the CAPEX for a typical WEC system is approximately 3,100 k€/MW, which is over 1.7 times higher than that of an offshore wind turbine at 1,789 k€/MW. DG technologies promise to reduce this high CAPEX by eliminating complex mechanical PTOs. However, the economic case hinges on a critical trade-off with OPEX, which is dominated by the potential cost of material replacement due to limited fatigue life.

Sensitivity analyses conducted by Guo et al. [130] reveal that the LCOE is highly sensitive to several key parameters. When the discount rate decreases from 11% to 6%, the LCOE reduces from $160/MWh to $102/MWh, representing approximately a 36% reduction. Similarly, extending device lifetime significantly impacts economic viability, with projections showing that achieving deployment levels above 2 GW could reduce the LCOE to between EUR 113-226/MWh by 2030. The study further demonstrates that under optimal combinations of reduced CAPEX/OPEX (by 75%), improved array arrangements, and increased annual energy production (by 12% to 55%), the target LCOE of $0.30/kWh becomes achievable. For DG technologies specifically, material fatigue life emerges as the dominant sensitivity parameter, as it directly affects both device lifetime and replacement costs within the OPEX.

In the context of this study, the CAPEX values used for the DG–WEC case refer primarily to the device and PTO costs, and do not explicitly include site-specific BoP items such as foundations, moorings, or grid connection infrastructure. This device-level focus is consistent with the techno-economic framework often adopted for early-stage wave energy concepts [132]. When interpreted alongside existing LCOE sensitivity studies, which show that realistic variations in CAPEX (approximately ±20% to 40%), OPEX (approximately ±10% to 30%), and capacity factor (approximately ±10% to 20%) can lead to large shifts in LCOE [130], it becomes clear that improving material durability to reduce replacement- and maintenance-related OPEX is at least as important as lowering the initial equipment CAPEX for DG–WEC systems and is therefore a decisive factor in achieving a competitive LCOE.

Therefore, the key to achieving a competitive LCOE for DG technologies depends less on incremental efficiency gains and more on a fundamental breakthrough in material durability.

### Emerging dielectric materials

Advanced dielectric composites now offer significantly improved energy densities and durability. Notably, barium titanate-doped silicones and fluorinated elastomers have achieved relative permittivity values exceeding 12 while maintaining low viscoelastic loss—a substantial improvement over traditional PDMS formulations [5]. Novel architectures such as 3D separated MWCNT–silicone matrices exhibit both high dielectric constants and structural integrity under cyclic loading [21].

Simultaneously, DFGs have benefited from the development of high-dielectric ester fluids and tunable electrode–membrane interfaces that support bistable behavior and improved energy capture efficiency. The use of Peano-HASEL-inspired architectures has improved modeling precision and structural responsiveness in low-frequency marine conditions.

### System-level integration and engineering challenges

Beyond advancements in materials, the transition of DG technologies to practical ocean energy systems hinges on solving critical system-level engineering challenges. A primary concern is the system-level integration of these soft, compliant generators with conventional rigid offshore hardware. The hydrodynamic coupling between a deforming generator and a floating platform or mooring system is highly complex; the platform’s motion drives the generator, but the generator’s viscoelastic response simultaneously influences the platform’s dynamics. Optimizing this interaction to maximize energy capture while ensuring stability is a formidable modeling and control problem [88]. The ultimate challenge in this domain is ensuring survivability under storm conditions, which is fundamentally a material science problem rooted in fatigue life.

To properly contextualize this durability challenge, a systematic comparison with established marine industries is necessary. An offshore wind turbine blade, for example, is designed for a fatigue life in the range of 10^8^ to 10^9^ loading cycles to guarantee a 25-year operational lifespan [133]. In stark contrast, even the best-performing DEGs currently demonstrate fatigue lives in the range of 10^6^ to 10^7^ cycles under laboratory conditions [63]. This 1- to 2-order-of-magnitude shortfall illustrates that material durability is not an incremental engineering problem but a fundamental scientific barrier that must be overcome for DG technology to be considered a reliable, long-term solution.

A parallel challenge exists in the power electronics required to condition the unique high-voltage, variable-capacitance output of DG systems. The reliability of these circuits is a known vulnerability in the harsh marine environment, with high humidity and salt spray increasing failure rates [134]. A critical trade-off must be made in the comparison of different conditioning circuit strategies. Simpler self-priming circuits offer greater autonomy and potential robustness, which is advantageous for remote deployments. However, they often operate at lower efficiency. In contrast, more complex strategies like buck-boost or multilevel converters can achieve higher conversion efficiencies and offer more sophisticated control, but their increased component count inherently introduces more potential failure points, reducing system reliability [5]. Developing robust, marine-hardened power conditioning units that are both highly efficient and fault-tolerant is a crucial research gap that remains to be addressed.

**Fig. 5.**
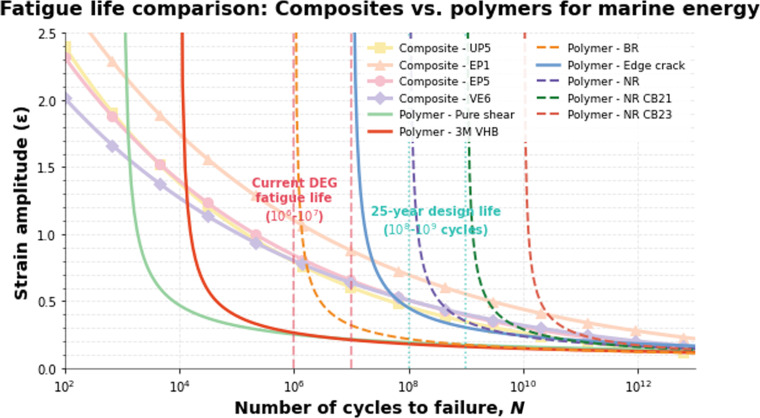
Fatigue life comparison (S–N curve) of composites and polymers for marine energy applications. The data for polymers and composites are adapted from [29,135], respectively.

As demonstrated in Fig. 5, the fatigue performance of various materials is plotted. These data come from leading research in the field [29,135]. For soft polymers and DEs used in DEGs, available S–N data show that fatigue life is governed by strain-dependent viscoelastic damage, the Mullins effect, and crack growth. There is no clear high-cycle linear region or fatigue limit in the conventional sense. At strain amplitudes relevant for DEG energy harvesting, lifetimes are typically limited to about 10^4^ to 10^7^ cycles, corresponding to the current DEG fatigue window. These lifetimes remain highly sensitive to the chosen failure criterion and operating conditions [63]. By contrast, the glass-fiber and carbon-fiber composites used in offshore wind blades exhibit S–N behavior that can be described reliably by power-law relations. This S–N behavior can be incorporated into design approaches based on Miner’s rule and multiaxial fatigue models, allowing 10^8^ to 10^10^ cycles at comparable strain levels and hence typical 20- to 25-year design lives [136]. This contrast highlights that, despite similar nominal cycle counts, DEG polymers currently lag behind mature composite blade materials in both high-cycle fatigue performance and the maturity of available fatigue models.

### Challenges in scaling-up

The reliability of power electronics is a key factor in determining the feasibility of wave energy conversion systems. Because the output power of wave energy devices is highly random and unstable, efficient rectifiers and grid-connected interfaces, as well as sophisticated control strategies and filtering methods, are crucial to ensuring power output quality and grid compatibility [137]. Existing power electronics experience a significant increase in failure rates when exposed to long-term marine environments with high humidity and salt spray. At the system level, the scaling challenges of power electronics primarily manifest in the complex coupling of environmental adaptability, heat dissipation and power density, as well as system redundancy and maintenance. Corrosion, moisture, and temperature cycling in marine environments place higher demands on component lifespan and reliability, necessitating the development of more corrosion-resistant packaging and redundant designs. Furthermore, as device power levels increase, heat dissipation and thermal management issues become more severe, directly impacting long-term stable operation [138]. Large-scale offshore operations require power electronics systems to possess fault tolerance and online monitoring capabilities to reduce maintenance costs and extend service life. The long-term stable operation of power electronics systems in offshore environments remains a major challenge hindering the large-scale application of DEG technology [134]. These systems must operate with high reliability in the corrosive and humid marine environment. A critical trade-off exists between different circuit strategies: Simpler, self-priming circuits may offer greater autonomy but at the cost of efficiency, while more complex multi-level converters promise higher efficiency but introduce more potential failure points. Developing robust and efficient power conditioning units tailored for DG systems is therefore essential for delivering stable, grid-compliant power from the ocean.

When it comes to ensuring uniformity and consistency in large-area thin-film materials, local defects or uneven thickness can significantly reduce not only the dielectric breakdown strength but also the device lifetime [29]. Currently, most experiments are still limited to small-scale samples. When scaling up to large-area manufacturing, maintaining stable mechanical and electrical properties becomes a key scalability challenge [5]. This issue not only involves the controllability of material preparation but also is closely related to process repeatability and quality inspection capabilities. Therefore, a systematic research framework is needed to integrate the multi-level challenges from material design, preparation process, to failure mechanism analysis [139]. DEs have demonstrated excellent performance in actuator and energy harvesting applications, but there is a lack of scalable engineering solutions for large-scale production, especially in terms of how to avoid defect accumulation in continuous processes and maintain long-term consistency [140]. Therefore, from a scalability perspective, future research should systematically focus on process optimization, online monitoring technologies, and evaluation methods coupled with long-term device reliability, providing a practical path for the transition from laboratory samples to industrial applications [139].

Environmental qualification testing is essential for validating DG system performance under realistic offshore conditions. Table 11 summarizes key marine aging effects based on standard protocols, including temperature cycling (IEC 60068-2-14), UV exposure (ASTM G154), and salt fog testing (ASTM B117). These tests simulated the corrosive and fatigue-accelerating effects of the marine environment on encapsulation, support structures, electrode films, and wire connectors. Polymer-based materials and composite structures exhibited significant performance degradation in these tests. If these aging effects are ignored during design and scale-up, the lifespan and reliability of engineered devices are often systematically overestimated [141]. In addition to material performance limitations, logistics and installation during offshore deployment pose key obstacles to scale-up. Flexible structures are susceptible to additional mechanical damage during transportation and installation, and their service reliability under long-term marine corrosion and complex load conditions lacks systematic verification and evaluation [98]. Compared to small-scale prototypes, large-scale deployments significantly rely on transport vessels, installation equipment, port conditions, and weather windows, introducing additional uncertainties and economic risks [142]. Furthermore, operation and maintenance costs for offshore energy conversion systems typically account for 20% to 30% of the total lifecycle cost, a proportion that is likely to rise further with large-scale deployment, significantly impacting overall economic feasibility [143]. In this context, reliable remote monitoring, condition monitoring, and fault diagnosis technologies are crucial to maintaining long-term stable system operation.

**Table 11.** Summary of marine environmental aging effects on dielectric elastomer materials

Stressor	Material	Degradation effects	Performance impact
Temperature cycling	Silicone (PDMS)	Microcrack formation	15–25% breakdown strength reduction [118]
	VHB 4910	Permanent set	30–40% mechanical loss increase [119]
UV radiation	VHB acrylic	Photo-oxidation	40% tensile strength loss [3]
	Silicone rubber	Surface hardening	20% surface modulus increase [5]
Salt fog/spray	Carbon electrode	Ionic contamination	50% surface resistivity reduction [117]
	Silicone membrane	Salt crystal formation	15–20% breakdown strength reduction [126]
Cycling	VHB 4910	Water absorption	46% breakdown field reduction [127]
Combined aging	Silicone DEG	Synergistic effects	60–70% lifetime reduction [119]

### Hybrid architectures and system-level integration

The integration of DEGs and DFGs into hybrid systems represents a critical step toward maximizing energy harvesting across diverse wave profiles. Hybrid platforms leverage the high adaptability of DEGs with the mechanical robustness of DFGs, enabling more resilient and efficient energy conversion systems. New modular configurations, such as segmented membranes and multi-mode fluid chambers, support redundancy and localized fault isolation, ensuring continued operation in the event of component failure.

Additionally, the development of self-powered modules with integrated charge pumps and fault-tolerant electronics has enabled autonomous operation without external power sources. Such systems are especially attractive for remote offshore deployments where grid connectivity is limited or infeasible.

### Manufacturing and scalability

Scalable manufacturing methods, particularly roll-to-roll processing of elastomeric films and additive manufacturing of microfluidic channels, have reduced the unit cost of DG components by up to 68% while preserving sub-millimeter tolerances [8]. Advances in machine learning-guided material dispersion and robotic assembly have further improved batch-to-batch consistency and reduced human error in fabrication.

Efforts are also underway to improve the environmental sustainability of DG systems. For instance, biodegradable NRs and recyclable thermoplastics are being explored as alternatives to thermoset silicones, which currently require energy-intensive pyrolysis for disposal.

### Challenges and commercialization roadmap

Despite substantial progress, several challenges remain. Material fatigue and electrode degradation continue to limit the service life of DEGs, especially under ultralow-frequency excitation. Environmental degradation, particularly in the form of microplastic release from elastomer surfaces, poses additional ecological risks. These issues must be mitigated through innovations in protective coatings and self-healing materials.

To realize commercial deployment, the following roadmap is proposed:

• Short-term (1 to 3 years): Optimize DEG and DFG materials for energy density and cyclic stability; develop standardized and industry-accepted testing protocols and performance benchmarks for long-term reliability.
• Mid-term (3 to 7 years): Deploy hybrid DEG–DFG prototypes in controlled offshore testbeds; validate survivability models and integrate with energy storage and grid-tied systems using marine-hardened power electronics.
• Long-term (7 to 10 years): Achieve mass production and certification of DG modules; establish pilot DG farms for utility-scale wave energy harvesting, with a focus on demonstrating a competitive LCOE.

## Conclusion

This review has synthesized the working principles, material advancements, and comparative performance of DEGs and DFGs, arriving at a key insight: The primary barrier to commercialization is not a matter of incremental efficiency, but the fundamental challenge of achieving long-term engineering reliability and durability in the harsh marine environment. While the potential of this technology to revolutionize wave energy is immense, realizing it requires a strategic shift in the research paradigm—from demonstrating what is possible in the laboratory to proving what is durable, reliable, and economical in the sea. To advance the field, future efforts must be concentrated on 3 core pillars: material innovation to close the critical durability gap, system-level integration to ensure survivability and robust power conversion, and scalable manufacturing to achieve long-term economic viability. As detailed in the roadmap, successfully navigating these challenges in a phased approach is essential for transitioning DG technology from a promising academic concept into a crucial component of the global clean energy portfolio, ultimately contributing to carbon reduction and the sustainability of coastal communities.

## Data Availability

All data supporting the findings of this study are available from the corresponding author upon reasonable request.
